# Diabetes in pregnancy and infant adiposity: systematic review and meta-analysis

**DOI:** 10.1136/archdischild-2015-309750

**Published:** 2016-05-26

**Authors:** Karen M Logan, Chris Gale, Matthew J Hyde, Shalini Santhakumaran, Neena Modi

**Affiliations:** Section of Neonatal Medicine, Chelsea and Westminster Hospital Campus, Imperial College London, London, UK

**Keywords:** Neonatology, Obesity, Endocrinology, Outcomes research

## Abstract

**Objective:**

Maternal glycaemia and anthropometry-derived newborn adiposity are strongly correlated. The children of mothers with diabetes are at greater risk of adverse metabolic health, and increased adiposity is a plausible mediator. We undertook a systematic review and meta-analysis to compare adiposity in infants of diabetic mothers (IDM) and infants of mothers without diabetes (NIDM).

**Design:**

We identified observational studies reporting adiposity in IDM and NIDM. We searched references, traced forward citations and contacted authors for additional data. We considered all body composition techniques and compared fat mass, fat-free mass, body fat % and skinfold thickness. We used random effects meta-analyses and performed subgroup analyses by maternal diabetes type (type 1, type 2 and gestational) and infant sex. We examined the influence of pre-pregnancy body mass index (BMI) and conducted sensitivity analyses.

**Results:**

We included data from 35 papers and over 24 000 infants. IDM have greater fat mass than NIDM (mean difference (95% CI)): 83 g (49 to 117). Fat mass is greater in infants of mothers with gestational diabetes: 62 g (29 to 94) and type 1 diabetes: 268 g (139 to 397). Insufficient studies reported data for type 2 diabetes separately. Compared with NIDM, fat mass was greater in IDM boys: 87 g (30 to 145), but not significantly different in IDM girls: 42 g (−33 to 116). There was no attenuation after adjustment for maternal BMI.

**Conclusions:**

IDM have significantly greater adiposity in comparison with NIDM. These findings are justification for studies to determine whether measures to reduce infant adiposity will improve later health.

**What is already known on this topic?**

- Offspring of mothers with diabetes have greater risks of adverse metabolic sequelae in later life.
- The underlying mechanisms are unclear but increased infant adiposity is a plausible mediator.
- A strong association has been demonstrated between maternal glycaemia and infant adiposity using indirect (anthropometry-derived) techniques.

**What this study adds?**

- This study quantifies the overall difference in adiposity between infants of mothers with and without diabetes derived from all body composition techniques.
- Maternal diabetes is associated with higher fat mass, body fat % and skinfold thickness in infancy.
- In subgroup analyses of studies providing sex-specific data, adiposity was higher in infants of diabetic mothers compared with NIDM boys but not girls.

## Introduction

Diabetes in pregnancy is increasing^1^ ^2^ and currently affects up to 5% of women in the UK. Approximately 87.5% of cases are gestational diabetes mellitus (GDM), 7.5% type 1 diabetes (T1D) and 5% type 2 diabetes (T2D).^3^ The offspring of mothers with diabetes have greater risks of adverse metabolic sequelae in childhood and later life^4–8^ and risks appear to be additional to genetic predisposition.^8–11^

The underlying mechanisms are unclear but increased infant adiposity is a plausible mediator. Adiposity in childhood and adult life is associated with T2D and cardiovascular disease^12^ ^13^ and we have previously shown that maternal diabetes in pregnancy is associated with an increased offspring body mass index (BMI) z-score in childhood.^4^ BMI is limited as an index of adiposity as it reflects both fat and lean mass and infants have large variations of body fat for a given BMI.^14^ The Hyperglycaemia and Adverse Pregnancy Outcome (HAPO) study identified a strong association between maternal glycaemia and infant anthropometry-derived adiposity.^15^ However, using more direct techniques to measure body composition in infants of diabetic mothers (IDM), the findings are inconsistent^16–21^ and many studies have been small with limited power. The magnitude of the difference in adiposity between IDM and NIDM derived from all body composition techniques has not been quantified.

We conducted a systematic review and meta-analysis to summarise available evidence of the impact of maternal diabetes on infant adiposity. Secondary objectives were to distinguish the effect of type of maternal diabetes and infant sex, which were not reported by the HAPO group.^15^ Sex-specific differences have previously been described in relation to maternal glycaemia.^17^ It has been suggested that associations between maternal hyperglycaemia and offspring outcome may be explained by confounding from maternal overweight.^22^ Therefore to establish whether maternal diabetes had independent effects on infant adiposity, we also performed analysis following adjustment for maternal pre-pregnancy BMI.

## Methods

### Literature search

We undertook a systematic review of published observational studies reporting adiposity in IDM and NIDM following MOOSE (meta-analyses and systematic reviews of observational studies) guidelines. We registered the protocol (see online supplementary file 1) on PROSPERO.^23^ We considered T1D, T2D and GDM as exposures. We planned to evaluate data from infants (ie, <1 year) and children (ie, 1–18 years). As a large quantity of data was obtained, we chose to summarise all infant data in one review and to perform a separate analysis for children. We searched in PubMed for studies published before 1 February 2014, without language restrictions, using the search strategy detailed (see online supplementary figure S1).

10.1136/fetalneonatal-2015-309750.supp1 Supplementary data

10.1136/fetalneonatal-2015-309750.supp2 Supplementary data

We excluded review articles after searching reference lists. Relevant studies were identified from either abstract or full paper. We searched reference lists of retrieved papers and attempted to trace forward citations. Where measures of adiposity were mentioned but not published, maternal diabetes status was unclear or mean and SD values were not provided, we contacted authors for additional data. If no response was received to two requests, or the author was unable to provide data, we excluded the study.

### Data extraction and analysis

Information on study populations, exposure, outcome, results and covariates was extracted and checked by a second author. Study quality was examined independently by three authors using a modified Newcastle–Ottawa Quality Assessment Scale (see online supplementary file 2).

We examined the association between maternal diabetes and each of the following variables independently in infants: fat mass, fat-free mass, body fat %, triceps and subscapular skinfold thickness. We used RevMan 5 (5.2), inverse variance and random effects methods as all studies were observational. We presented differences between groups as pooled mean difference (95% CI).

10.1136/fetalneonatal-2015-309750.supp3 Supplementary data

We presented body composition results derived from skinfold thickness or other techniques as separate subgroups and as a pooled result. Raw skinfold thickness data were presented separately. Where studies only reported different types of diabetes separately, we calculated pooled means and SD for all types combined. Where studies provided adjusted results, we performed separate meta-analyses of adjusted and unadjusted data.

We used forest plots to illustrate results and funnel plots to investigate publication/small study bias.^24^ If funnel plots showed asymmetry, we performed Egger's test.

### Between-study heterogeneity

We assessed heterogeneity using the χ^2^ test for the Q statistic and calculated I^2^, an estimate of the proportion of variance due to between-study heterogeneity.

We investigated potential sources of heterogeneity according to prespecified subgroups (type of maternal diabetes, body composition technique and study quality). We checked whether conclusions differed when only high-quality studies were analysed by conducting a meta-analysis restricted to studies with a high modified Newcastle–Ottawa score (5 out of 5).

We also performed subgroup analysis by infant sex and large for gestational age/macrosomic infants. We performed a separate meta-analysis of all studies providing results adjusted for maternal BMI. We calculated the mean difference in maternal pre-pregnancy BMI between mothers with and without diabetes for each individual study and plotted this against the mean difference in infant fat mass or body fat %. If the graphs suggested that studies with larger differences in maternal BMI had larger differences in offspring adiposity, we would have performed a meta-regression.

## Results

### Literature search

We identified 431 papers, of which 45 matched inclusion criteria. We identified two additional studies from reference lists.^25^ ^26^ We contacted five authors for body composition or maternal diabetes data; two provided data.^27^ ^28^ Thirty-five papers remained in the systematic review, following exclusions (see online supplementary figure S1). Seven authors provided outcome means and SD on request,^15^ ^17^ ^18^ ^20^ ^27^ ^29^ ^30^ and final meta-analysis data were available from 27 studies. We analysed neonatal (ie, infants <4 weeks old) measurements separately. We report body composition data in table 1. We also present skinfold thickness data (see online supplementary table S1) and describe all included studies (see online supplementary table S2).

**Table 1:** Body composition data in infants of mothers with and without diabetes from individual studies included in the systematic review using (A) skinfolds and (B) other techniques

Study	Study groups	Age	Fat mass (g)				Fat-free mass (g)				% Fat mass			
			Controls		IDM		Controls		IDM		Controls		IDM	
*(A) Studies using skinfold thickness*														
Aman *et al*^31^	Controls: 28 IDM: 28 (18 T1D, 10 GDM)	<48 h	500 (200)		700 (200)		3100 (400)		3400 (400)		13.5 (3.5)		16.4 (3.2)	
Brunner *et al*^28^	Controls: 152 (82 males) IDM: 9 (all GDM) (three males)	3–5 days	Males	Females	Males	Females	Males	Females	Males	Females	Males	Females	Males	Females
			482 (146)	485 (138)	653 (290)	438 (94)	3029 (352)	2939 (342)	3505 (433)	2647 (276)	13.5 (2.7)	14.0 (2.8)	15.2 (4.1)	14.1 (1.9)
			Pooled		Pooled		Pooled		Pooled		Pooled		Pooled	
			483 (142)		509 (195)		2988 (350)		2933 (528)		13.7 (2.8)		14.5 (2.6)	
Enzi *et al*^25^	Controls: 17 IDM: 25 (8 T1D, 17 GDM)	Birth	386 (91)		606 (185)		Author contacted—no further data available				12.2 (2.1)		18.1 (6.1)	
McFarland *et al*^42^	Controls: 58 (40 males) IDM: 16 (eight males) (12 GDM, 4 pre-existing)	<24 h	762 (243)		1012 (292)		3519 (236)		3282 (267)		17.7		23.5	
Metzger (HAPO)^15^	Controls: 16 097 IDM: 3082 (all GDM)	<72 h	375 (159)		424 (177)		2866 (311)		2928 (334)		11.2 (3.53)		12.2 (3.70)	
Schaefer-Graf *et al*^26^	Controls: 190 (92 males) IDM: 150 (all GDM) (66 males)	<48 h	381 (179)		433 (171)		Authors contacted—no further data available							
Zhao *et al*^21^	Controls: 284 (139 males) IDM: 160 (all GDM) (90 males)	<48 h	Males	Females	Males	Females	Males	Females	Males	Females	Males	Females	Males	Females
			475 (61)	484 (84)	579 (61)	588 (57)	2800 (105)	2764 (109)	2695 (121)	2674 (133)	14.4 (1.1)	14.7 (2.2)	17.2 (0.5)	17.9 (0.8)
			Pooled		Pooled		Pooled		Pooled		Pooled		Pooled	
			480 (74)		585 (59)		2784 (109)		2685 (127)		14.7 (1.9)		17.8 (0.8)	
*(B) Studies using techniques other than skinfold thickness*														
Au *et al*^18^	Controls: 532 (284 males) IDM: 67 (all GDM) (28 males)	<48 h	Males	Females	Males	Females	Males	Females	Males	Females	Males	Females	Males	Females
			306 (184)	358 (172)	268 (181)	275 (181)	3033 (350)	2874 (314)	3017 (351)	2717 (268)	8.4 (4.3)	10.3 (4.2)	7.4 (4.2)	8.4 (4.7)
			Pooled		Pooled		Pooled		Pooled		Pooled		Pooled	
			331 (180)		272 (180)		2959 (342)		2846 (338)		9.3 (4.3)		7.9 (4.5)	
Brumbaugh *et al*^19^	Controls: 13 (seven males) IDM: 12 (all GDM) (eight males)	16.3±2.3 days (1–3 weeks)	Author contacted—no further data available								13.1 (5.0)		14.7 (3.0)	
Catalano *et al*^16^	Controls: 220 (119 males) IDM: 195 (all GDM) (100 males)	<72 h	Males	Females	Males	Females	Males	Females	Males	Females	Males	Females	Males	Females
			352 (197)	374 (200)	463 (200)	407 (210)	3044 (428)	2894 (369)	3071 (369)	2847 (411)	9.9 (4.6)	10.9 (4.5)	12.7 (4.4)	12.0 (4.9)
			Pooled		Pooled		Pooled		Pooled		Pooled		Pooled	
			362 (198)		436 (206)		2975 (408)		2962 (405)		10.4 (4.6)		12.4 (4.6)	
Durnwald *et al*^44^	Controls: 52 (26 males) IDM: 50 (all GDM, but LGA babies) (31 males)	<48 h	563 (206)		662 (163)		3557 (310)		3400 (314)		13.5 (4.5)		16.2 (3.3)	
Hammami *et al*^43^	Controls: 36 IDM: 11 (nine GDM, one T1D, one T2D, but all LGA babies)	1.8 (1.0) days	905 (248)		1242 (177)		3393 (213)		3343 (143)		20.4 (4.5)		26.4 (2.7)	
Lee *et al*^20^	Controls: 324 (160 males) IDM: 25 (13 GDM, 9 T1D, 3 T2D) (11 males)	<60 h	Males	Females	Males	Females	Males	Females	Males	Females	Males	Females	Males	Females
			323 (161)	351 (183)	565 (193)	542 (229)	2935 (437)	2752 (383)	3050 (479)	2891 (419)	9.5 (3.6)	10.8 (4.2)	15.5 (4.0)	15.2 (4.4)
			Pooled		Pooled		Pooled		Pooled		Pooled		Pooled	
			337 (173)		552 (210)		2843 (420)		2961 (444)		10.2 (4.0)		15.4 (4.2)	
Lingwood *et al* 17	Controls: 77 (41 males) IDM: 84 (all GDM) (42 males)	<6 days	Males	Females	Males	Females	Males	Females	Males	Females	Males	Females	Males	Females
			353 (149)	346 (179)	400 (194)	427 (191)	3189 (294)	2880 (266)	2943 (314)	2835 (340)	9.76 (3.55)	10.39 (4.58)	11.6 (4.4)	12.7 (4.1)
			Pooled		Pooled		Pooled		Pooled		Pooled		Pooled	
			350 (162)		413 (192)		3045 (320)		2889 (329)		10.05 (4.05)		12.1 (4.3)	

GDM, gestational diabetes mellitus; HAPO, Hyperglycaemia and Adverse Pregnancy Outcome; LGA, glycated haemoglobin; T1D, type 1 diabetes; T2D, type 2 diabetes.

### Fat mass

**Figure 1:**
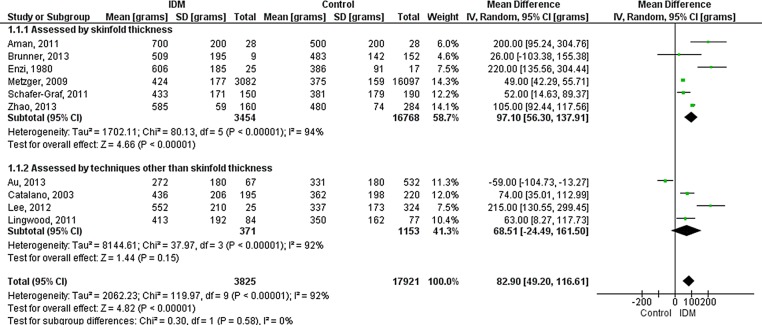
Forest plot (random effects analysis) comparing fat mass (g) in IDM and NIDM (all types of diabetes).

Ten studies provided unadjusted data for IDM (all types) and NIDM. Six studies derived fat mass from skinfold thickness,^15^ ^21^ ^25^ ^26^ ^28^ ^31^ three studies used air displacement plethysmography (ADP)^17^ ^18^ ^20^ and one study used total body electrical conductivity (TOBEC).^16^ Fat mass was higher in IDM (overall 83 g (49 to 117); p<0.00001) (figure 1). The pooled mean difference of 83 g represents 22% greater fat mass in IDM in comparison with the mean fat mass of NIDM across all studies.

### Fat-free mass

**Figure 2:**
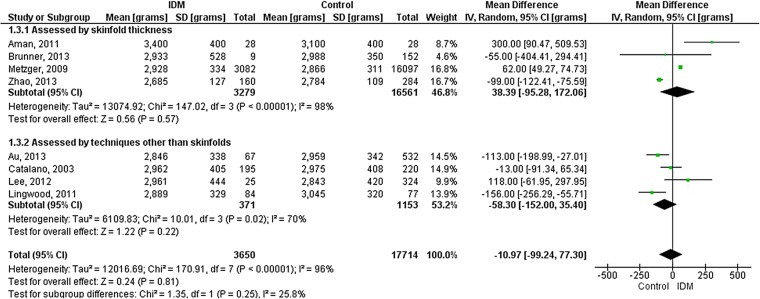
Forest plot (random effects analysis) comparing fat-free mass (g) in IDM and NIDM (all types of diabetes).

Eight studies provided unadjusted data. Four studies used skinfold thickness,^15^ ^21^ ^28^ ^31^ three studies used ADP^17^ ^18^ ^20^ and one study used TOBEC.^16^ There was no significant difference in fat-free mass between IDM and NIDM (overall −11 g (−99 to 77); p=0.81) (figure 2).

### Body fat %

**Figure 3:**
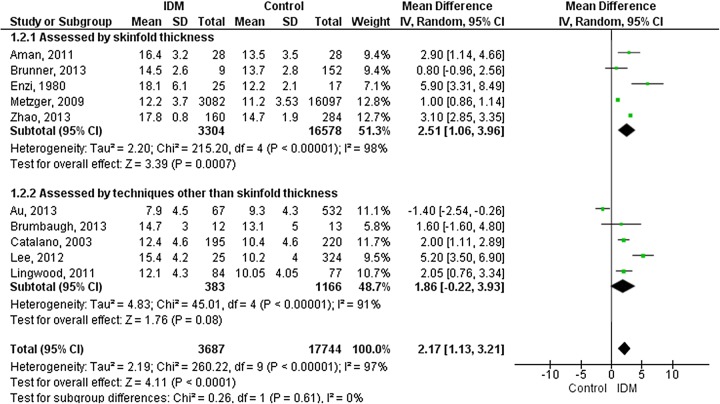
Forest plot (random effects analysis) comparing body fat % in IDM and NIDM (all types of diabetes).

Ten studies provided unadjusted data. Five studies used skinfold thickness,^15^ ^21^ ^25^ ^28^ ^31^ four studies used ADP^17–20^ and one study used TOBEC.^16^ Body fat % was higher in IDM (overall 2.2% (1.1% to 3.2%); p<0.0001) (figure 3). There was no evidence of funnel plot asymmetry for any outcome (see online supplementary figures S2–S4).

### Triceps and subscapular skinfold thicknesses

Raw and unadjusted triceps and subscapular skinfold thicknesses were reported in 17 studies.^15^ ^16^ ^21^ ^27–40^ Both were higher in IDM (0.52 mm (0.37 to 0.68) and 0.81 mm (0.56 to 1.05), respectively; p<0.00001) (see online supplementary figures S5–S6).

### Subgroup analyses

#### Types of maternal diabetes

##### Gestational diabetes mellitus

Ten studies provided body composition data in infants of mothers with and without GDM.^15–19^ ^21^ ^25^ ^26^ ^28^ ^31^ Infants of mothers with GDM had higher fat mass (62 g (29 to 94); p=0.0002) (see online supplementary figure S7) and body fat % than NIDM (1.7% (0.7% to 2.8%); p=0.002) (see online supplementary figure S8), but fat-free mass was not significantly different (−23 g (−116 to 70); p=0.62) (see online supplementary figure S9). Raw and unadjusted skinfold data were reported in 12 studies.^15^ ^16^ ^21^ ^27–31^ ^33^ ^37–39^ Infants of mothers with GDM had higher triceps (0.47 mm (0.27 to 0.66); p<0.00001) and subscapular skinfolds (0.69 mm (0.37 to 1.02); p<0.0001). Heterogeneity remained high for all outcomes (χ^2^ p<0.05, I^2^>92%).

##### Type 1 diabetes

Two studies presented separate body composition data^25^ ^31^ and one presented skinfold thickness data^34^ in infants of mothers with T1D. Fat mass (268 g (139 to 397), p<0.0001) and body fat % (5.3% (−0.1% to 10.7%), p=0.05) were higher in IDM (see online supplementary figures S7 and S8). Heterogeneity was significant for body fat % (χ^2^ p=0.0005, I^2^=92%), but not for fat mass (χ^2^ p=0.11, I^2^=61%).

Maternal diabetes type accounted for 89% of the variation in fat mass and 39% of the variation in body fat %, although the difference for body fat % was not statistically significant (indicated by test for subgroup differences in forest plots).

##### Type 2 diabetes

No study provided separate data for infants of mothers with T2D.

#### Infant sex

One study reported sex-specific data^17^ and we received data from nine additional authors.^16^ ^18^ ^20^ ^21^ ^27^ ^28^ ^30^ ^38^ ^41^ Six studies provided unadjusted body composition data in IDM and NIDM girls and boys.^16–18^ ^20^ ^21^ ^28^ IDM girls had lower fat-free mass than NIDM girls (−85 g (−152 to −17); p=0.01), but fat mass (42 g (−33 to 116); p=0.27) and body fat % (1.5% (−0.4% to 3.4%); p=0.13) were not significantly different. IDM boys had higher fat mass (87 g (30 to 145); p=0.003) and higher body fat % (2.3% (1.0% to 3.7%); p=0.0008) than NIDM boys, but fat-free mass was not significantly different (−49 g (−150 to 52); p=0.34). Heterogeneity was not detected between male and female subgroups for any outcome (χ^2^ p>0.05, I^2^=0%). Of note, in this subgroup analysis, when sexes were combined, the results for fat mass and body fat % were similar to the overall analyses, but fat-free mass was significantly lower in IDM (−76 g (−123 to −29), p=0.002).

Six studies reported raw and unadjusted skinfold data in IDM and NIDM girls and boys.^16^ ^21^ ^27^ ^28^ ^30^ ^38^ IDM girls had greater triceps and subscapular skinfolds (p<0.05) than NIDM girls. IDM boys had greater triceps and subscapular skinfolds (p<0.001) than NIDM boys.

#### Large for gestational age/macrosomic infants

Three studies provided separate body composition data in large for gestational age/macrosomic IDM and NIDM.^42–44^ In IDM, fat mass was higher (220 g (62 to 379); p=0.006) and fat-free mass was lower (−140 g (−246 to −34); p=0.009).

### Heterogeneity

Heterogeneity was statistically significant with between-study differences accounting for >90% of variation throughout (χ^2^ and I^2^ values in forest plots). The following additional potential sources of heterogeneity were investigated.

#### Type of technique

Studies assessing adiposity using skinfold thickness were compared with those using other techniques (figures 1–3). Technique accounted for none of the variation in fat mass or body fat % and 26% of the variation in fat-free mass, though there were no statistically significant differences between the groups. Furthermore, heterogeneity remained high within the technique subgroups.

#### Study quality

Only one study achieved a highly modified Newcastle–Ottawa score; a separate analysis was not possible.^15^

#### Adjusted analyses

One study provided data adjusted for a number of confounders; a separate analysis was not possible.^15^

### Maternal pre-pregnancy BMI

We included studies providing maternal BMI measured pre-pregnancy or during pregnancy as these are closely correlated. Four studies adjusted body composition for BMI obtained pre-pregnancy^17^ ^18^ ^21^ or at the time of glucose tolerance test (GTT).^15^ A meta-analysis of the unadjusted data showed greater fat mass (73 g (27 to 119), p=0.002) in IDM, but differences in body fat % (1.2% (−0.3% to 2.8%), p=0.11) and fat-free mass (−72 g (−188 to 45), p=0.23) were not statistically significant. The results were similar with adjusted data (fat mass 64 g (12 to 115), p=0.02; body fat % 1.2% (−0.3% to 2.6%), p=0.11; fat-free mass −64 g (−182 to 54), p=0.29).

Eight studies reported maternal pre-pregnancy BMI^16–19^ ^21^ ^26^ ^28^ or BMI at the time of GTT.^15^ Plots of mean difference in maternal BMI between mothers with and without diabetes against mean difference in infant fat mass and body fat % showed no evidence of a relationship between increasing maternal BMI and increasing infant adiposity (see online supplementary figures S10–S11).

## Discussion

We have shown that maternal diabetes is associated with significantly higher fat mass, body fat % and skinfold thickness in infancy. We summarised data acquired using a range of body composition techniques, from 35 papers and over 24 000 infants. We followed a preregistered public protocol, with the aim of reducing reporting bias^23^ and included studies from ethnically diverse countries.

The main limitation was the high degree of study heterogeneity. We investigated potential sources by sensitivity analysis, namely study quality, body composition technique and maternal diabetes type. Subgroup analysis of study quality was not possible as only one high-quality study was identified.^15^ This reflects the small and observational nature of the majority of studies included. Subgroup analyses of data derived from skinfold thickness and other techniques revealed no significant differences between subgroups and heterogeneity was high irrespective of the technique used. The majority of studies included offspring of mothers with GDM and the overall findings were mainly reflective of this group. Significant heterogeneity remained in the subgroup analysis of studies of mothers with GDM. The variable definition and treatment of GDM among studies are likely contributing factors. Adiposity was significantly higher in infants of mothers with T1D, but there were insufficient studies to perform separate meta-analyses for T2D. Metabolic effects of exposure to diabetes in utero appear to be similar regardless of diabetes type,^8^ ^45^ ^46^ but the effect on infant adiposity warrants further investigation. Though we identified a high degree of study heterogeneity, the consistency of findings led to greater confidence in the conclusions. IDM also had greater fat mass than NIDM within the subgroup of large for gestational age/macrosomic infants. This finding supports an additional risk to metabolic health in these infants following exposure to maternal diabetes.^47^

A further strength was the provision of additional sex-specific data from many authors, enabling exploration of sex-specific effects of maternal diabetes. Fat mass and body fat % were statistically higher in IDM compared with NIDM boys but not girls. Boys grow more quickly and may be more vulnerable to glycaemic fluctuation. Lingwood *et al*^17^ found maternal fasting blood glucose to be the major predictor of infant body fat in boys but not in girls. Regnault *et al*^48^ found sex-specific associations of maternal glucose tolerance with childhood adiposity, but not fat-free mass. In our analysis of fat-free mass, there was very wide heterogeneity. We found significantly lower fat-free mass in IDM in some subgroups, including studies which provided sex-specific estimates (in girls but not in boys), but could not explain the heterogeneity between studies. The studies reported had limited power for sex-specific differences to be adequately explored. We recommend that future studies are powered to detect sex-specific effects.

The relationship between maternal diabetes in pregnancy and offspring adiposity has been examined in two previous systematic reviews but neither assessed effects in infancy and in both BMI was used as a measure of overweight.^4^ ^49^ We previously found an association between maternal diabetes and childhood BMI z-score, which was attenuated in studies adjusting results for maternal pre-pregnancy BMI.^4^ In contrast, Kim *et al*^49^ found no statistically significant relationship between GDM and offspring BMI in the majority of studies, but did not perform a meta-analysis.

The rising prevalence of GDM in low/middle-income countries is strongly linked to increasing maternal obesity. The HAPO group found that maternal GDM and, to a lesser extent, maternal obesity were independently associated with newborn adiposity with their combination having the greatest impact.^50^ We performed a separate examination of studies that adjusted for maternal BMI; fat mass remained significantly higher in IDM, supporting an independent effect of maternal diabetes. This is also supported by sibling comparison studies which show that children born after their mother developed diabetes as opposed to before have higher systolic blood pressure, glycated haemoglobin, BMI and nearly four times the odds of developing T2D.^9–11^

We have shown that maternal diabetes is associated with greater infant adiposity. As fat mass appears to track from infancy into childhood, this may be a harbinger of longer term risks to health.^51^ ^52^ A randomised controlled trial of GDM treatment showed reduced neonatal adiposity.^53^ However, little association was found between maternal glycaemia and offspring obesity at age 2 years in HAPO participants in Belfast (one of 15 study centres),^54^ nor have follow-up studies shown reduced early childhood obesity following treatment of GDM,^55^ ^56^ though intriguingly female offspring had lower fasting glucose concentrations.^55^ In conclusion, published evidence identifies maternal diabetes as a risk factor for offspring adiposity. Whether this is a causal mediator for the well-recognised risks to the later health of IDM remains to be established.
