# Supplementary material for: Diabetes in pregnancy and infant adiposity: systematic review and meta-analysis
Source: Arch Dis Child Fetal Neonatal Ed. 2016 May 26;102(1):F65–72. doi: 10.1136/archdischild-2015-309750 (PMC5256410; doi:10.1136/archdischild-2015-309750)
Supplement: Supplementary data [file fetalneonatal-2015-309750supp1.pdf]

UNIVERSITY of York
Centre for Reviews and Dissemination

NHS
National Institute for Health Research

PROSPERO International prospective register of systematic reviews

The diabetic pregnancy and offspring adiposity in infancy and childhood: a systematic review and meta-analysis

Karen Logan, Shalini Santhakumaran, Matthew Hyde, Chris Gale, Neena Modi

Citation

Karen Logan, Shalini Santhakumaran, Matthew Hyde, Chris Gale, Neena Modi. The diabetic pregnancy and offspring adiposity in infancy and childhood: a systematic review and meta-analysis. PROSPERO 2012:CRD42012003004 Available from http://www.crd.york.ac.uk/PROSPERO_REBRANDING/display_record.asp?ID=CRD42012003004

Review question(s)

Do offspring born to mothers with diabetes (ODM) in pregnancy have altered body composition in infancy and childhood, when compared to controls from non-diabetic pregnancies?

Searches

((Gestation* OR Pregnan*)

AND

(Diabet*))

AND

((body composition[MESH]) OR (body composition [All fields]) OR (bioelectrical impedance[MESH]) OR (bioelectrical impedance [All fields]) OR (total body electrical conductivity) OR (Tomography, X-Ray Computed[MESH]) OR (Tomography, X-Ray Computed [All fields]) OR (absorptiometry, photon[MESH]) OR (absorptiometry, photon [All fields]) OR (total body potassium) OR (magnetic resonance imaging[MESH]) OR (magnetic resonance imaging [All fields]) OR (air-displacement plethysmography) OR (isotope dilution) OR (skinfold) OR (skin fold))

AND

((Child*) OR (infan*) OR (neonat*) OR (offspring) OR (adolescen*) OR (ped*) OR (paed*))

Types of study to be included

• There are no restrictions on the types of study design eligible for inclusion. All study types reporting body composition (fat mass, fat free mass, % body fat or skinfold thickness) in ODM and control children will be included.

• Where population based cohorts are identified by the search strategy but outcomes are not reported on the basis of maternal diabetes, attempts will be made to extract this information for these studies by contacting the data custodian, in order to reduce effects of publication bias.

Condition or domain being studied

• Diabetes is a common pregnancy complication, affecting up to 5% of pregnancies in the UK (1) and the number of pregnancies complicated by pre-existing or gestational diabetes is rising.

• Exposure to diabetes in utero has been shown to influence long-term metabolic health, irrespective of type of maternal diabetes. We have previously published meta-analyses demonstrating greater body mass index and BP in ODM in childhood compared to healthy controls. Greater adiposity has been implicated as an early marker of adverse metabolic health. It appears to occur even in newborn ODM with appropriate weight for gestational age and may

track from infancy into childhood.

• It is unclear whether maternal diabetes type alters body composition in offspring.

• The relative influences of maternal glucose tolerance and maternal obesity on offspring body composition and BMI are unclear.

• Studies show a possible male predisposition to adiposity in groups at increased risk of type 2 diabetes.

• We therefore propose to conduct a systematic review and meta-analysis of available data on measures of body composition (fat mass, fat free mass, % body fat or skinfold thickness) in infants and children, in relation to exposure to maternal diabetes in pregnancy.

1. NICE guideline 63. Management of diabetes and its complications. In, wwwniceorguk/CG063. 2009. Epub 2010/01/08. 37. Jahrestagung der Osterreichischen Diabetes Gesellschaft : "Management des Diabetes und seiner Komplikationen - eine Standortbestimmung." Salzburg, 19.-21. November 2009.

2. Bell R, Bailey K, Cresswell T, Hawthorne G, Critchley J, Lewis-Barned N. Trends in prevalence and outcomes of pregnancy in women with pre-existing type I and type II diabetes. BJOG. 2008;115(4):445-52.

3. Dabelea D, Snell-Bergeon JK, Hartsfield CL, Bischoff KJ, Hamman RF, McDuffie RS. Increasing prevalence of gestational diabetes mellitus (GDM) over time and by birth cohort: Kaiser Permanente of Colorado GDM Screening Program. Diabetes Care. 2005;28(3):579-84.

4. Dabelea D. The predisposition to obesity and diabetes in offspring of diabetic mothers. Diabetes care. 2007;30 Suppl 2:S169-74.

5. Philipps LH, Santhakumaran S, Gale C, Prior E, Logan KM, Hyde MJ, et al. The diabetic pregnancy and offspring BMI in childhood: a systematic review and meta-analysis. Diabetologia. 2011;54(8):1957-66. 6. Aceti A, Santhakumaran S, Logan KM, Philipps LH, Prior E, Gale C, et al. The diabetic pregnancy and offspring blood pressure in childhood: a systematic review and meta-analysis. Diabetologia. 2012.

7. Increased fetal adiposity: a very sensitive marker of abnormal in utero development. Am J Obstet Gynecol. 2003;189(6):1698-704.

8. Catalano PM, Farrell K, Thomas A, Huston-Presley L, Mencin P, de Mouzon SH, et al. Perinatal risk factors for childhood obesity and metabolic dysregulation. Am J Clin Nutr. 2009;90(5):1303-13.

9. Hyperglycemia and Adverse Pregnancy Outcome (HAPO) Study: associations with neonatal anthropometrics. Diabetes. 2009;58(2):453-9.

10. Hillier TA, Pedula KL, Schmidt MM, Mullen JA, Charles MA, Pettitt DJ. Childhood obesity and metabolic imprinting: the ongoing effects of maternal hyperglycemia. Diabetes care. 2007;30(9):2287-92.

11. Lingwood BE, Henry AM, d'Emden MC, Fullerton AM, Mortimer RH, Colditz PB, et al. Determinants of body fat in infants of women with gestational diabetes mellitus differ with fetal sex. Diabetes care. 2011;34(12):2581-5.

12. Thomas EL, Parkinson JR, Hyde MJ, Yap IK, Holmes E, Dore CJ, et al. Aberrant adiposity and ectopic lipid deposition characterize the adult phenotype of the preterm infant. Pediatr Res. 2011;70(5):507-12.

Participants/ population

Outcomes will be evaluated in infants (i.e. <1 year old) and children (i.e. 1-18 years) born to mothers with and without diabetes in pregnancy. If possible, studies in children will be divided into age group categories; 1-4, 5-10 and 11-18 years, reflecting alterations in fat mass during childhood.

13. Veldhuis JD, Roemmich JN, Richmond EJ, Rogol AD, Lovejoy JC, Sheffield-Moore M, et al. Endocrine control of body composition in infancy, childhood, and puberty. Endocr Rev. 2005;26(1):114-46.

Intervention(s), exposure(s)
Maternal diabetes in pregnancy

Comparator(s)/ control
Normal glucose tolerant pregnancies

Context
The review will include all identified studies.

Outcome(s)
Primary outcomes
Difference in fat mass, fat free mass, % body fat and skinfold thickness between infants and children born to mothers with and without diabetes in pregnancy.

Secondary outcomes
• Difference in outcomes between infants and children born to normal glucose tolerant mothers and mothers with T1D, T2D and GDM

• Difference in effect size in boys and girls

• Difference in outcomes following adjustment for maternal pre-pregnancy BMI

• Differences with age of offspring

• Differences based on method of body composition measurement

Data extraction, (selection and coding)
• Titles and abstracts (where available) of studies identified from the initial searches will be independently assessed by two reviewers for possible inclusion. The full texts of all potentially eligible studies that have been identified will then be appraised by two assessors independently. Where there is disagreement over eligibility for inclusion, this will be referred to a meeting of all authors. Only studies where both maternal diabetes status and offspring body composition are reported in the paper will be included in the systematic review and meta-analysis.

• Data will be extracted independently onto a pre-piloted data collection form. The following data will be extracted for each study: type of study (cross sectional/longitudinal; retrospective/prospective); country of study; study population, setting and demographics of research subjects (gender, age at study, outcome studied, maternal BMI); details of exposure (type of diabetes, diagnostic criteria, treatment); inclusion and exclusion criteria; recruitment and study completion rates; outcome measurements; and adjusted analyses performed (yes/no and factors adjusted for). Missing data will be requested from study authors.

Risk of bias (quality) assessment
• Review level Bias:

Bias from small study effects will be assessed using funnel plots and Egger's test; where asymmetry is evident on the funnel plot a trim and fill analysis will be used. Possible causes for asymmetry, other than publication bias (e.g. between-study heterogeneity), will also be considered.

• Study level bias:

A modified Newcastle-Ottawa scale will be used to assess methodological quality of each individual study (carried out independently by 2 assessors, discrepancies to be resolved by group discussion). A subgroup analysis of the studies scoring at least 5 out of 7 stars will be conducted. Ranked forest plots (on the basis of the score out of 7 that each study achieves) will be produced for primary outcomes, to allow assessment of study quality on effect size.

Strategy for data synthesis
Data collected will be systematised into a table. All studies identified in the systematic review will be included in the

table whether or not they are included in the meta-analysis. A narrative description of these studies will be produced. Where studies are comparable on the basis of study population, intervention and outcome measure, the results will be pooled in a random-effects meta-analysis, with mean differences, 95% confidence intervals and two sided p-values. Heterogeneity will be assessed using the Chi-squared test for the Q statistic and calculation of I-squared, an estimate of the proportion of variance due to between-study heterogeneity. The random effects method will be used as the default as all studies are observational, however where there is no evidence of heterogeneity (p value from Chi-squared test >0.05) and there are at least 5 studies, a fixed effects meta-analysis will also be performed to check the sensitivity of the conclusions to method choice. Where between-study heterogeneity is very high (I-squared over 80%) and is not explained through differences in study characteristics we will re-consider whether quantitative data synthesis is appropriate.

Analysis of subgroups or subsets

Meta-regression and subgroup analysis will be used to explore sources of heterogeneity arising from study characteristics (type of diabetes, gender, age, method of body composition measurement and study quality). Data from studies adjusting for confounders will be synthesised in a separate meta-analysis to the unadjusted data.

Dissemination plans

The results of this review will be presented at meetings of relevant societies and interest groups. It will be written up for peer-reviewed publication.

Contact details for further information

Professor Neena Modi

Neonatal medicine, floor 4, lift bank D, Chelsea and Westminster Hospital Campus, Imperial College London, SW10 9NH

n.modi@imperial.ac.uk

Organisational affiliation of the review

Imperial College London

http://www1.imperial.ac.uk/departmentofmedicine/divisions/infectiousdiseases/paediatrics/neonatalmedicine/

Review team

Dr Karen Logan, Imperial College London
Ms Shalini Santhakumaran, Imperial College London
Dr Matthew Hyde, Imperial College London
Dr Chris Gale, Imperial College London
Professor Neena Modi, Imperial College London

Details of any existing review of the same topic by the same authors

None

Anticipated or actual start date

03 September 2012

Anticipated completion date

15 January 2013

Funding sources/sponsors

KL received support from Chelsea and Westminster Health Charity and is currently funded by a fellowship from Action Medical Research; CG received support by Chelsea and Westminster Health Charity. SS is funded through a National Institute of Health Research programme grant held by Professor Modi. No external funding was received specifically for this work.

Conflicts of interest
None known

Language
English

Country
England

Subject index terms status
Subject indexing assigned by CRD

Subject index terms
Adiposity; Diabetes, Gestational; Humans; Prenatal Exposure Delayed Effects;

Stage of review
Ongoing

Date of registration in PROSPERO
28 September 2012

Date of publication of this revision
28 September 2012

Stage of review at time of this submission	Started	Completed
Preliminary searches	Yes	No
Piloting of the study selection process	Yes	No
Formal screening of search results against eligibility criteria	No	No
Data extraction	No	No
Risk of bias (quality) assessment	No	No
Data analysis	No	No

PROSPERO
International prospective register of systematic reviews
The information in this record has been provided by the named contact for this review. CRD has accepted this information in good faith and registered the review in PROSPERO. CRD bears no responsibility or liability for the content of this registration record, any associated files or external websites.
