# Supplementary material for: Diabetes in pregnancy and infant adiposity: systematic review and meta-analysis
Source: Arch Dis Child Fetal Neonatal Ed. 2016 May 26;102(1):F65–72. doi: 10.1136/archdischild-2015-309750 (PMC5256410; doi:10.1136/archdischild-2015-309750)
Supplement: Supplementary data [file fetalneonatal-2015-309750supp_figures.pdf]

Study	Study groups	Age	Triceps skinfold thickness (mm)				Subscapular skinfold thickness (mm)			
			Controls		IDM		Controls		IDM	
Aman, 2011 [31]	Controls: 28<br>IDM: 28<br>(18 T1D, 10 GDM)	<48 hours	5.3 (1.1)		6.7 (1.4)		4.8 (1.1)		6.3 (1.8)	
Brans, 1983 [57]	Controls: 52<br>IDM: 61<br>(49 GDM, 12 pre-existing)	<12 hours	Skinfold thickness (sum of triceps and subscapular skinfolds) Control: 7.73 (1.98) IDM 8.87 (1.58)							
Brumbaugh, 2013 [19]	Controls 13 (7 male)<br>IDM 12 (all GDM) (8 male)	16.3±2.3 days [1-3 weeks]	Skinfold thickness (sum of triceps and subscapular skinfolds) Control: 9.88 (1.96) IDM: 11.73 (1.30)							
Brunner, 2013 [28]	Controls: 152<br>(82 male)<br>IDM: 9 (all GDM)<br>(3 male)	3-5 days	**Males**	**Females**	**Males**	**Females**	**Males**	**Females**	**Males**	**Females**
			4.6 (0.9)	4.7 (0.8)	5.5 (1.6)	5.0 (0.6)	4.4 (0.9)	4.6 (1.0)	4.9 (1.4)	4.6 (0.3)
			**Pooled**		**Pooled**		**Pooled**		**Pooled**	
			4.7 (0.9)		5.2 (0.9)		4.5 (1.0)		4.7 (0.8)	
Buhling, 2012 [29]	Controls: 142<br>IDM: 30 (all GDM)	<72 hours	4.8 (1.5)		4.6 (0.9)		4.1 (0.97)		4.3 (1.1)	
Catalano, 2003 [16]	Controls: 220 (119 male)<br>IDM: 195 (all GDM) (100 male)	<72 hours	**Males**	**Females**	**Males**	**Females**	**Males**	**Females**	**Males**	**Females**
			4.1 (1.1)	4.2 (0.8)	4.8 (1.1)	4.6 (1.1)	4.4 (1.2)	4.7 (1.2)	5.5 (1.4)	5.3 (1.4)
			**Pooled**		**Pooled**		**Pooled**		**Pooled**	
			4.2 (1.0)		4.7 (1.1)		4.6 (1.2)		5.4 (1.4)	
Clarson, 1989 [58]	Controls: 11<br>IDM: 30<br>(11 GDM, 19 pre-existing)	<48 hours	Data for sum of 8 skinfolds (midbiceps, midtriceps, subscapular and suprailiac on both sides)<br>Control: 29.0 (5.0) IDM: 33.6 (7.3)							
Enzi, 1980 [25]	Controls: 17<br>IDM: 25<br>(8 T1D, 17 GDM)	Birth	Data for sum of 4 skinfolds (subcostal, subscapular, triceps, crural)<br>Control: 17.8 (2.9), IDM: 25.5 (6.6)							
Greco, 2003 [32]	Controls: 16<br>ODM: 15<br>(GDM, T1D, T2D)	<24 hours	4.1 (0.1)		4.7 (0.1)		3.8 (0.1)		4.7 (0.1)	
Hollingsworth, 1991 [33]	Controls: 211<br>ODM: 70 (all GDM)	Newborn - time not stated	3.8 (0.3)		4.3 (0.9)		4.0 (0.4)		5.0 (1.1)	
Krishnaveni, 2005 [30]	Controls: 545 (266 male)<br>IDM: 41 (all GDM) (15 male)	<72 hours	**Males**	**Females**	**Males**	**Females**	**Males**	**Females**	**Males**	**Females**
			4.1 (0.9)	4.2 (0.9)	4.8 (0.8)	5.2 (1.2)	4.4 (0.9)	4.5 (1.0)	5.1 (1.0)	5.4 (1.1)
			**Pooled**		**Pooled**		**Pooled**		**Pooled**	
			4.2 (0.9)		5.1 (1.1)		4.4 (0.9)		5.3 (1.1)	

McFarland, 1998 [42]	Controls: 58 (40 male)<br>IDM: 12 (12 GDM, 4 pre-existing) (8 male)	<24 hours	5.4 (1.2)	7.5 (1.5)	5.4 (1.3)	6.8 (2.0)
Metzger (HAPO), 2009 [15]	Controls: 16097<br>IDM: 3082 (all GDM)	<72 hours	4.08 (0.86)	4.42 (0.99)	4.17 (0.95)	4.59 (1.13)
Mohamed, 2010 [59]	Controls: 20 (10 male)<br>IDM: 40 (all T1D) (26 male)	Birth	1.3	2.32 (0.49)	0.48	1.43 (0.55)
Nasrat, 1997 [60]	Controls: 501 (236 male)<br>IDM: 50 (40 GDM, 10 T2D) (29 male)	<12 hours	3.12	3.26	3.4	3.8
Nelson, 2007 [34]	Controls: 19<br>IDM: 56<br>(all T1D)	Birth	6.0 (2.4)	8.0 (3.1)	5.6 (2.0)	7.4 (2.1)
Ng, 2004 [61]	Controls: 40<br>IDM: 80<br>(68 GDM, 12 T1D)	Birth	TR and SS presented as median and IQR in control, diet treated and insulin treated diabetes groups. No statistically significant differences between the groups.			
Petersen, 1990 [62]	Controls: 16<br>IDM: 20<br>(5 GDM, 15 pre-existing diabetes)	<48 hours	Data for sum of 5 skinfolds only; quadriceps, pectoralis, biceps, subscapular and triceps<br>Control: 21.2 (3.6), IDM: 26.8 (5.2)			
Rossi, 2000 [35]	Control: 13<br>IDM: 6 (GDM and T1D)	Birth	4.76 (0.28)	5.78 (1.5)	4.47 (2.2)	5.78 (1.25)
Simmons, 1995 [36]	Control: 125<br>(61 male)<br>IDM: 35 (34 GDM, 1 T2D) (14 male)	<24 hours	4.2 (0.8)	4.5 (1.0)	5.3 (1.3)	6.3 (2.0)
Sletner, 2013 [27]	Control: 457<br>(230 male)<br>IDM: 65 (all GDM)<br>(32 male)	<72 hours	**Males** 4.36 (0.99)<br>**Females** 4.44 (1.01)<br>**Pooled** 4.40 (1.00)	**Males** 4.70 (1.21)<br>**Females** 4.72 (1.05)<br>**Pooled** 4.71 (1.12)	**Males** 4.19 (0.90)<br>**Females** 4.34 (1.11)<br>**Pooled** 4.26 (1.02)	**Males** 4.78 (1.33)<br>**Females** 4.90 (1.33)<br>**Pooled** 4.84 (1.32)
Stevenson, 1991 [37]	Control: 20<br>IDM: 13 (all GDM)	<72 hours	4.3 (0.8)	5.0 (1.1)	No SS measurement taken	
Verma, 1991 [63]	750 newborns<br>(males: 413)<br>No control/ODM numbers	<48 hours	3.73 (0.88)	3.92 (0.83)	3.75 (0.83)	3.99 (0.85)
Vohr, 1995 [38]	Controls: 143 (76 male)<br>IDM: 119 (all GDM)<br>(59 male)	Birth	**Males** 3.9 (1.0)<br>**Females** 4.2 (1.2)<br>**Pooled** 4.1 (1.0)	**Males** 4.0 (1.0)<br>**Females** 4.0 (1.2)<br>**Pooled** 4.1 (1.1)	**Males** 4.5 (1.3)<br>**Females** 4.8 (1.3)<br>**Pooled** 4.6 (1.3)	**Males** 4.6 (1.4)<br>**Females** 4.8 (1.5)<br>**Pooled** 4.7 (1.5)

<table>
<tr><td>Westgate, 2006
[39]</td><td>Controls: 61
IDM: 136
(108 GDM, 28 T2D)</td><td><24 hours</td><td colspan="2">4.4 (1.0)</td><td colspan="2">5.1 (1.2)</td><td colspan="2">4.4 (1.0)</td><td colspan="2">5.6 (1.7)</td></tr>
<tr><td>Whitelaw, 1977
[64]</td><td>Controls: 45 (AGA infants)
IDM: 18</td><td><48 hours
1 year</td><td colspan="8">Data for sum of 8 skinfolds (biceps, triceps, subscapular and suprailiac on both sides)
Birth: Control: 28.3 (4.2) IDM: 41.3 (10.9) 1 year: Control: 26.9 IDM: 24.0</td></tr>
<tr><td>Wurster, 1984
[40]</td><td>Control: 10 (7 male)
IDM: 10 (GDM and pre-existing)
(3 male)</td><td><48 hours
2 weeks
1 month</td><td colspan="2">5.0 (1.5)
5.0 (1.0)
5.8 (1.2)</td><td colspan="2">5.9 (1.5)
6.2 (1.1)
6.3 (0.8)</td><td colspan="2">5.2 (1.6)
6.0 (1.1)
6.6 (1.2)</td><td colspan="2">6.6 (1.9)
6.6 (1.2)
6.9 (1.1)</td></tr>
<tr><td rowspan="4">Zhao, 2013
[21]</td><td rowspan="4">Controls: 284 (139 male)
IDM: 160 (all GDM)
(90 male)</td><td rowspan="4"><48 hours</td><td>Males</td><td>Females</td><td>Males</td><td>Females</td><td>Males</td><td>Females</td><td>Males</td><td>Females</td></tr>
<tr><td>4.1 (0.1)</td><td>4.3 (0.2)</td><td>5.1 (0.2)</td><td>5.3 (0.3)</td><td>3.9 (0.1)</td><td>4.0 (0.2)</td><td>5.1 (0.3)</td><td>5.2 (0.2)</td></tr>
<tr><td colspan="2">Pooled</td><td colspan="2">Pooled</td><td colspan="2">Pooled</td><td colspan="2">Pooled</td></tr>
<tr><td colspan="2">4.4 (0.3)</td><td colspan="2">5.2 (0.4)</td><td colspan="2">4.0 (0.2)</td><td colspan="2">5.2 (0.3)</td></tr>
</table>

Supplementary, table 1: Triceps and subscapular skinfold thickness in infants of mothers with and without diabetes from individual studies included in the systematic review

Author, year, reference	Study details	Diabetes definition and treatment	Body composition measurement	Details of adjusted analyses
Aman, 2011 [31]	PC; single centre, Sweden; GDM and T1D.<br>Control: term infants of mothers with negative screening for GDM in pregnancy matched for gestational age and mode of delivery.<br>Blinding not stated.<br>Newcastle-Ottawa score 4.	GDM defined as 2h glucose >11 mmol/l on 75g OGTT.<br>All GDM mothers treated with diet and multiple pre-meal insulin injections. BG self-monitored daily and mothers monitored twice monthly in clinic with treatment adjusted to blood glucose.	Examined by single study nurse. Harpenden caliper used for skinfold thickness. Body fat mass calculated from birth weight, birth length and flank skinfold thickness using equation proposed by Catelano.	No
Au, 2013 [18] (additional data provided by authors)	Cross-sectional; single centre, Sydney, Australia; GDM.<br>Control: singleton, term infants with no congenital anomalies whose mothers had normal glucose tolerance.<br>Exclusions: pre-existing diabetes.<br>Babies admitted to NICU > 2 days excluded.<br>Blinding not stated.<br>Newcastle-Ottawa score 3.	Diagnosis of GDM based on the Australasian Diabetes In Pregnancy Society (ADIPS) criteria at time of study. Dietary and physical activity advice given and BG monitored four times daily. 3rd trimester pre-and post-prandial mean BGs obtained or progress notes of BGs reviewed. HbA_{1c} levels obtained. Insulin commenced when glycemic targets not met on dietary adjustment.	Neonatal body fat %, fat mass, and fat-free mass assessed at birth using air displacement plethysmography. Adjusted for infancy (refs 4, 6, 7 in text).	Associations between GDM status and body composition investigated using linear regression unadjusted and adjusted for potential confounders: gestational age, infant sex, maternal age, pre-pregnancy BMI, gestational weight gain, parity, smoking, ethnicity, and hypertension.
Brans, 1983 [57]	PC; single centre, USA: GDM, T1D and T2D. 22 of 61 infants of mothers with diabetes were macrosomic (birth weight >90th percentile).	GDM diagnosed by 3hr 100g OGTT in 3rd trimester (at least 2 BGs exceeding thresholds; fasting 105 mg/dL, 1h 195	Skinfold thickness measured on the left side using a Harpenden caliper by the same investigator. Sum of measurements to the	No

	Control: infants of mothers with no diabetes (antenatal fasting BG <100 mg/dL) and no family history of diabetes. 28 of 52 control infants were macrosomic.<br>Blinding not stated.<br>Newcastle-Ottawa score 2.	mg/dL, 2h 165 mg/dL, 3h 145 mg/dL).<br>All GDM women treated with diet and 23 treated with insulin. All described as good control and only 10 had raised HbA1C.	nearest 0.05mm presented.	
Brumbaugh, 2013 [19]	PC; multi-centre, USA; GDM (all had BMI >30 kg/m2)<br>Control: term infants of mothers with negative OGTT at 24-28 weeks of pregnancy and BMI <25 kg/m^2.<br>Exclusions: pre-existing diabetes and IUGR infants.<br>Blinding not stated.<br>Newcastle-Ottawa score 2.	GDM diagnosed by 3hr OGTT using Carpenter and Coustan criteria.<br>Treatment not described.	Triceps and subscapular skin fold thickness obtained in triplicate by single investigator with a Lange caliper. Total fat mass and fat-free mass measured with air displacement plethysmography. Adjusted for infancy (refs 22, 23 in text). MRI for subcutaneous and intra-abdominal fat.	No
Brunner, 2013 [28] (additional data provided by authors)	Munich population study, Germany; GDM. Data from a follow up of an RCT of LCPUFA supplementation in pregnancy (controls only presented);<br>Control: singleton term infants of mothers with normal glucose tolerance and pre-pregnancy BMI 18-30.<br>Exclusions: pre-existing diabetes.<br>Blinding to maternal diabetes status not described (but not the aim of the study).<br>Newcastle-Ottawa score 3.	For meta-analysis the authors provided the data by maternal diabetes status. GDM diagnosis established from data provided by the women´s gynaecologists. GDM screening was not routine at the time of the study and not all women underwent screening.	Skinfold thickness was measured by trained research assistants in triplicate using a Holtain caliper on the left side and the mean measurement used. Body fat % was calculated using Weststrate's method.	No
Buhling, 2012 [29]	PC; single centre, Germany; GDM.<br>Control: infants of mothers with negative screening or no risk factors for GDM.<br>Blinding not stated.<br>Newcastle-Ottawa score 3.	GDM diagnosed by OGTT and ADA criteria. Treatment not described.	Skinfold thicknesses measured using a single observer with a single measurement and a Holtain caliper.	No adjustments of skin fold thickness results.

Catalano, 2003 [16] (additional data provided by authors)	PC; single centre, USA; GDM.<br>Control: singleton term infants of mothers with 1h 50g glucose screen <135mg/dl or normal 3h 100g OGTT.<br>Exclusions: infants <2000g.<br>Blinding not stated.<br>Newcastle-Ottawa score 2.	GDM diagnosed by National Diabetes Data Group criteria.<br>Diet controlled unless glycaemic target not achieved (fasting glucose <100 mg/dl or 2h postprandial glucose <120mg/dL), then insulin controlled.<br>67 women with GDM required insulin.	Total body electrical conductivity to assess fat free mass, fat mass and body fat %. 10 measurements taken and averaged.<br>Adjusted for infancy (ref 12 in text).<br>Skinfold thickness assessed using a Harpenden caliper. Anthropometric and TOBEC measurements performed by 1 of 3 examiners.	Unadjusted data presented and adjustments made for gestational age, maternal pregravid weight, weight at last antenatal visit, race, smoking status, and maternal and paternal height.
Clarson, 1989 [58]	PC; single centre, Canada; GDM and pre-existing diabetes.<br>Controls: term infants of mothers with normal glucose tolerance.<br>Blinding not stated.<br>Newcastle-Ottawa score 1.	GDM diagnosed by OGTT at the end of the 2nd trimester using O'Sullivan criteria. Insulin treatment given to all women with GDM from time of diagnosis.	Skinfold thickness measured using a Lange caliper.	No
Durnwald, 2004 [44]	PC; single centre, USA; LGA infants (>90th percentile for gestational age, race and sex) of GDM and non-diabetic mothers.<br>Control: LGA infants of mothers with either normal 1hr 50g OGTT or 3h 100g OGTT.<br>Blinding not stated.<br>Newcastle-Ottawa score 3.	GDM diagnosed by Carpenter and Coustan criteria. Diet controlled unless glycaemic target not achieved (fasting glucose <95 mg/dl or 2h postprandial glucose <120mg/dL), then insulin controlled. 26 of 50 mothers received insulin.	Total body electrical conductivity to assess fat free mass, fat mass and body fat %. 10 measurements taken and averaged.<br>Adjusted for infancy (ref 14 in text).<br>Skinfold thickness assessed using a Harpenden caliper. 92 body composition measurements by TOBEC and 10 by skinfolds.	Unadjusted data presented and stepwise regression analysis used with adjustment for maternal age, pre-pregnancy weight, gestational age and smoking history.

Enzi, 1980 [25]	PC; single centre, Italy; GDM and T1D.<br>Control: term infants of mothers with negative screening for GDM in pregnancy (100g OGTT).<br>Blinding not stated.<br>Newcastle-Ottawa score 1.	Pre-existing diagnosis of T1D or GDM diagnosed by 100g OGTT and National Data Group Criteria.<br>Dietary advice provided and insulin given in T1D group 3 times daily. T1D mothers also hospitalised 6 times during pregnancy for BG assessment.	Skinfold thickness assessed using a Harpenden caliper, and body fat mass calculated from anthropometric measurements by Dauncey's method (infant specific).	No
Greco, 2003 [32]	PC; single centre, Italy; GDM or pre-existing well-controlled (HbA1c 3.5-5.3%) diabetes.<br>Controls: singleton infants of primiparous Caucasian women with no relevant medical history and negative 28 week 100g OGTT, matched for age and pre-pregnancy weight.<br>Blinding not stated.<br>Newcastle-Ottawa score 2.	Diabetes classified using White Class. Intensive monitoring to achieve strict glucose control. Maternal pre-prandial and post-prandial BG assessment 3 times daily. HbA1c monitored every 4 weeks and insulin therapy altered on basis of BG monitoring and HbA1c.	Skinfold measurements taken from the left hand side by a single observer using calipers and the mean of 3 measurements calculated.	No
Hammami 2001 [43]	PC; single centre, USA; GDM or pre-existing diabetes. LGA (≥4000g and >90th percentile) v AGA. IDM in LGA group only. LGA babies presented.<br>Controls: infants of mothers with normal blood glucose screening or normal glucose tolerance test.<br>Blinding not stated.<br>Newcastle-Ottawa score 2.	Mothers with diabetes defined by National Diabetes Data Group criteria. 9 with GDM (7 on insulin), 1 with T2D and 1 with T1D (both on insulin).	DXA whole body scans performed with a single beam densitometer. Adjusted for infancy (ref 7 in text).	Unadjusted data presented. Regression analysis used to investigate weight, length, race and sex as influences of body composition in AGA infants.
Hollingsworth, 1991 [33]	PC; single centre, USA; GDM.<br>Control: infants of mothers with negative 1h 50g glucose screen in pregnancy.<br>Newcastle-Ottawa score 3.	GDM diagnosed by ≥2 abnormal values on 3h 100g OGTT using O'Sullivan criteria. Treatment not stated.	Skinfold thickness measured. No further detail given.	No

Krishnaveni, 2005 [30] (additional data provided by authors)	RC; single centre, India; GDM.<br>Controls: singleton infants of mothers with negative GDM screening.<br>Exclusions: pre-existing diabetes and SGA infants.<br>Blinding not stated.<br>Newcastle-Ottawa score 4.	GDM diagnosed following 100g 3h OGTT using Carpenter and Coustan criteria. All mothers treated with diet and 12 (29%) received insulin.	Skinfold thickness measured by one of four trained observers using Harpenden caliper.	No
Lee, 2012 [20] (additional data provided by authors)	PC; multi-centre, USA; singleton infants of uncomplicated pregnancies for development of adiposity reference ranges. Study included an additional cohort of 25 infants of mothers with GDM or pre-existing diabetes. Exclusions: pregnancies with poor menstrual dating and infants with congenital anomalies.<br>Blinding not stated.<br>Newcastle-Ottawa score 2.	The authors state "An important study limitation was the unavailability of precise diagnostic criteria for classifying diabetic pregnancies." T1D, T2D and GDM included. No treatment information discussed.	Percentage body fat and total body fat mass estimated using direct measurements of volume and body mass based on principles of whole body densitometry. Adjusted for infancy (ref 29 in text).	Unadjusted data supplied by authors. Data adjusted for newborn weight, gestational age, gender and ethnicity using multiple regression. Variables entered the model if p <0.05 and were removed if p >0.10.
Lingwood, 2011 [17] (additional data provided by authors)	PC; single centre, Australia; GDM.<br>Control: singleton term infants of mothers with normal glucose tolerance and BMI (18.5-25) at booking. Recruited in the same centre over a similar time period and assessed by the same methods but reported separately.<br>Exclusions: mothers with a history of maternal illness other than GDM and infants with congenital anomalies.<br>Newcastle-Ottawa score 2.	GDM diagnosis by 75g GTT (fasting ≥5.5 or 2h ≥8.0 mmol/L) and ADIPS criteria. Treated following ADIPS guidelines. Diet and activity advice. BG levels monitored 4x per day with targets of fasting ≤5.5 or 2h ≤8.0 mmol/L. Insulin commenced if necessary. Relatively well-controlled group of women with GDM (80% met both ADIPS targets above on average).	Body composition measured by air displacement plethysmography. Infant body fat % was computed from body density by software integral to the system. Age and sex-specific densities of fat-free mass were computed based on data of Fomon	No

Metzger (HAPO), 2009 [15] (additional data provided by authors)	PC; multi-centre, multinational study, ethnically diverse cohort involving 15 field centres; GDM. Medical caregivers blinded to maternal glucose levels unless following criteria met: fasting plasma glucose >5.8 mmol/L, 2hr OGTT >11.1 mmol/L or random glucose ≥8.9mmol/L. Singleton infants.<br>For purpose of meta-analysis authors grouped GDM and controls using more recent International Association of Diabetes in Pregnancy Study Groups (IADPSG) thresholds (FPG >5.1 mmol/L, 2hr OGTT >8.5 mmol/L.)<br>Blinding occurred as GDM diagnosed post Hoc.<br>Newcastle-Ottawa score 5.	GDM defined retrospectively using IADPSG thresholds. As women with GDM had BG levels below the pre-defined study diabetes thresholds, caregivers remained blinded to their values and no monitoring or treatment was provided.	Rigorous training and annual recertification in anthropometric measurements was established for research nurses and midwives. 2 skinfold measurements were taken using a Harpenden caliper and if results differed by >0.5mm, a 3rd was taken. Averages used. Fat mass calculated using equation by Catalano (infant specific). (4)	Authors provided unadjusted and adjusted data. Adjustments made for field centre, gestational age, maternal parity, neonatal gender and maternal BMI at the time of the OGTT.
McFarland, 1998 [42]	PC; single centre, USA; GDM and pre-existing diabetes. Macrosomic (>4000g) infants only.<br>Control: macrosomic infants of mothers with negative glucose screening during pregnancy.<br>Blinding not stated.<br>Newcastle-Ottawa score 2.	GDM diagnosed by National Diabetes Data Group criteria.<br>Diabetes treated by local protocol (ref 11 in text). BG monitored 7x/day.<br>Glucose control in group demonstrated to be poor (mean BG 125±34 mg/dL).	All skinfold measurements performed by one of two examiners. Estimation of body fat mass performed by Dauncey's method (infant specific).	No
Mohamed, 2010 [59]	PC; single centre, Egypt; T1D. Divided into macrosomic and non-macrosomic groups (> or < 4000g)<br>Control: term infants, <4000g of non-diabetic mothers, matched for age and sex. Glucose screening in pregnancy not discussed.<br>Exclusions: included infection, congenital or chromosomal abnormalities and metabolic disorders.	TID diagnosed pre-pregnancy. No further details.	Skinfold thickness measured using a skinfold caliper.	No

	Blinding not stated.<br>Newcastle-Ottawa score 2.			
Nasrat, 1997 [60]	PC; single centre, Saudi Arabia; GDM and pre-existing diabetes.<br>Control: singleton infants of mothers with negative glucose screening in pregnancy.<br>Blinding not stated.<br>Newcastle-Ottawa score 3.	GDM diagnosed following 100g 3h OGTT using NGGD criteria. 9 of 40 GDM and 0 of 10 T2D required insulin.	Skinfold thickness measured using a Harpenden caliper by one of 2 trained examiners.	No
Nelson, 2007 [34]	PC; multi-centre study, UK; T1D.<br>Control: infants of mothers with negative routine screening for gestational diabetes (national guidelines: http://www.sign.ac.uk/guidelines/fulltext/55/section8.html).<br>Blinding not stated.<br>Newcastle-Ottawa score 4.	T1D diagnosis and treatment not described.	Skinfold thickness at subscapular and triceps measured using a Holtain caliper by paediatricians using a centrally agreed protocol.	No
Ng, 2004 [61]	PC; single centre, Hong Kong; GDM and T1D.<br>Control: term infants admitted to neonatal unit with suspected perinatal infection but with negative cultures and normal CRP (<10mg/l). GDM screening not discussed.<br>Exclusions: infants with chromosomal or congenital abnormalities.<br>Blinding not stated.<br>Newcastle-Ottawa score 1.	T1D diagnosed prior to study.<br>GDM diagnosed by fasting glucose >5.5mmol/L or >8.0mmol/L 2h after 75g OGTT.<br>GDM treated with low energy diet (1800kcal/d) and T1D treated with daily insulin.	Skinfold thickness measured using a Holtain skinfold caliper.	No

Petersen, 1990 [62]	PC; single centre, Denmark; GDM and pre-existing diabetes. 3 groups of infants: SGA (<10th percentile), infants of mothers with diabetes and controls.<br>Control: term AGA infants of mothers with normal glucose tolerance.<br>Blinding not stated.<br>Newcastle-Ottawa score 1.	Maternal diabetes categorized by White classification. (GDM 5, pre-existing 15) Further details of diagnosis or treatment not given.	Skinfold thickness measured using a Harpenden caliper.	No
Rossi, 2000 [35] (Italian)	PC; single centre, Italy; GDM and T1D.<br>Control: women without diabetes.<br>Blinding not stated.<br>Newcastle-Ottawa score 2.	No information provided on the diagnosis and management of GDM. T1D treated with insulin.	Skinfold thickness measured. No further details provided.	No
Simmons, 1995 [36]	PC; two centres, New Zealand (Maori, Indian and Pacific Islanders); GDM or previous diet treated diabetes.<br>Controls: singleton term infants of non-diabetic mothers with no other medical illness and smoking <10 cigarettes/day.<br>Exclusions: mothers with pre-eclampsia and IUGR infants. European diabetic women excluded as numbers small.<br>Blinding not stated.<br>Newcastle-Ottawa score 3.	GDM diagnosed using O'Sullivan criteria. Women with diabetes maintained on 1800 cal diet. Those with continuing hyperglycaemia (fasting BG >5.5 mmol/L, 2h post prandial BG >6.5 mmol/L) despite therapy were treated with insulin. 15% required insulin.	Skinfold thickness measured using a Holtain paediatric caliper. All measures taken by one observer on the left side and the mean of 3 recordings was taken.	Data adjusted for neonatal sex and maternal ethnic group.
Schaefer-Graf, 2011 [26]	PC; single centre, Germany; GDM from previous study (ref 2 in paper) compared with controls.<br>Controls: singleton infants of mothers with normal 75g OGTT.	GDM diagnosed by 75g GTT using Carpenter and Coustan criteria (2 values above 5.0/10.0/8.6 mmol/l). Women given dietary advice and	Skinfold thickness measured at the flank and fat mass calculated by formula derived by Catalano (infant specific).	Unadjusted body composition data presented.

	Exclusions: infants <34 weeks or with congenital anomalies.<br>Blinding not stated.<br>Newcastle-Ottawa score 3.	performed self-monitoring of BG. Insulin therapy given based on BG levels or glycaemia plus fetal growth (randomised in initial trial). Equivalent outcomes in both groups of GDMs.		
Sletner, 2013 [27] (additional data supplied by authors)	Population-based cohort; multi-centre study, Norway; GDM. Authors separated skinfold results based on maternal GDM status for purpose of review.<br>Controls: infants of mothers with negative 75g screening OGTT at 28 weeks gestation.<br>Exclusions: pre-existing diabetes.<br>Blinding not stated.<br>Newcastle-Ottawa score 4.	GDM cases were diagnosed using WHO criteria. Women with FPG >7.0 mmol/l or 2h BG >9.0 mmol/l were referred to secondary care and those with 2h BG 7.8–9.0 mmol/l were referred to their GP after lifestyle advice had been given.<br>Authors also provided results by IADPSG criteria.	Measurements performed by specially trained personnel. Skinfolds measured using a Holtain caliper. Measurements performed twice to nearest 0.1 mm and means used.	Unadjusted data presented. Authors also provided data adjusted for maternal BMI.
Stevenson, 1991 [37]	PC; single centre, USA; GDM. AGA and LGA infants recruited.<br>Controls: term, AGA age infants.<br>Blinding not stated.<br>Newcastle-Ottawa score 1.	GDM diagnosed by glucose tolerance test using O'Sullivan and Mahan criteria. Dietary control then initiated.	Triceps skin fold measured by the same individual using a Lange caliper with mean taken of 3 separate measurements.	No
Verma, 1991 [63]	PC; single centre; India.<br>All infants delivered consecutively included. Preterms and SGA included. Maternal antenatal history including diabetes recorded.<br>Blinding not stated.<br>Newcastle-Ottawa score 3.	Maternal complications in the antenatal period, including diabetes recorded. No further details and numbers of infants of mothers with diabetes not given.	Skinfold thickness using a Harpenden caliper.	No

Vohr, 1995 [38] (additional data provided by authors)	PC; single centre, USA; GDM enrolled to include equal numbers of LGA and AGA babies (separated into LGA and AGA groups).<br>Controls: infants of mothers with negative 1h 50g glucose screen at 24-28 weeks, enrolled to include equal numbers of LGA and AGA babies.<br>Exclusions: infants with congenital anomalies or requiring intensive care.<br>Blinding not stated.<br>Newcastle-Ottawa score 3.	GDM diagnosed by 100g OGTT and Carpenter and Coustan criteria. Initial dietary advice and weekly BG monitoring aiming to keep fasting glucose <100mg/dl and all other values <120mg/dl. Insulin therapy recommended if these levels were exceeded.	Skinfolds measured by 1 of 2 trained research nurses, mean taken of 2 measurements using a Lange caliper.	No
Vohr, 1997 [41] (additional data provided by authors)	PC (subgroup of Vohr 1995); single centre, USA; GDM enrolled to include equal numbers of LGA and AGA babies (separated into LGA and AGA groups).<br>Controls: infants of mothers with negative 1h 50g glucose screen at 24-28 weeks, enrolled to include equal numbers of LGA and AGA babies.<br>Exclusions: infants with congenital anomalies or requiring intensive care.<br>Blinding not stated.<br>Newcastle-Ottawa score 2.	GDM diagnosed by 100g OGTT and Carpenter and Coustan criteria. Initial dietary advice and weekly BG monitoring aiming to keep fasting glucose <100mg/dl and all other values <120mg/dl. Insulin therapy recommended if these levels were exceeded.	Skinfolds measured by 1 of 2 research nurses, mean taken of 2 measurements using Lange caliper.	No
Westgate, 2006 [39]	PC; single centre, New Zealand (Maori and Pacific island mothers); GDM and T2D.<br>Control: infants of mothers with 1h 50g glucose screen <7.8mmol/L. Mothers invited using pseudo-random number generator against clinic lists and matched by ethnicity.<br>Blinding not stated.	T2D diagnosed prior to pregnancy. GDM diagnosed by fasting glucose ≥5.5mmol/L or 2h value ≥9.0. BG monitoring at home (5 tests daily), with insulin commenced if fasting BG >5.5 mmol/L on 2 occasions or post-prandial readings consistently ≥6.5 mmol/L.	Neonatal paediatrician or senior neonatal nurse assessed babies using a Harpenden caliper for skinfold thickness.	No

	Newcastle-Ottawa score 3.	Insulin pump used if daily dosage >200 units.		
Whitelaw, 1977 [64]	PC; single centre, UK; 5 groups of infants studied: SGA (<10th percentile), AGA (10th-90th percentile), LGA (>90th percentile), infants of mothers with diabetes and infants of obese mothers.<br>Control: AGA infants of mothers with normal glucose tolerance.<br>Newcastle-Ottawa score 2.	No details of diabetes type, diagnosis, or treatment.	Skinfolds measurements by a single observer using a Harpenden caliper.	No
Wurster, 1984 [40]	PC; single centre, USA; GDM or pre-existing diabetes.<br>Controls: infants of mothers without diabetes.<br>No blinding stated.<br>Newcastle-Ottawa score 2.	No details of diabetes diagnosis. Blood glucose monitored 4 times a day during pregnancy with an aim of maintaining pre-meal BG <100 mg/dL. Achieved in majority and women described as well-controlled diabetics.	Skinfold measurements taken on the left side of the body using a Harpenden caliper.	Unadjusted data presented and further subgroup analysis performed to confirm that infant sex was not an influence.
Zhao, 2013 [21] (Chinese) (additional data provided by authors)	PC; single centre, China; GDM v control in AGA (birth weight 10-90th percentile of local population for sex and gestation) infants.<br>Blinding not stated.<br>Newcastle-Ottawa score 3.	GDM diagnosis based on 1998 WHO diagnostic definition.	Anthropometric and skinfold measurements obtained and fat mass and fat-free mass calculated.	Unadjusted data presented and regressions performed of factors associated with differences in the anthropometric measurements but adjusted results not provided.

Supplementary, table 2: Studies included in the systematic review examining the association between intrauterine exposure to maternal diabetes and offspring adiposity in infants. GDM: gestational diabetes mellitus, T1D: type 1 diabetes, T2D: type 2 diabetes.

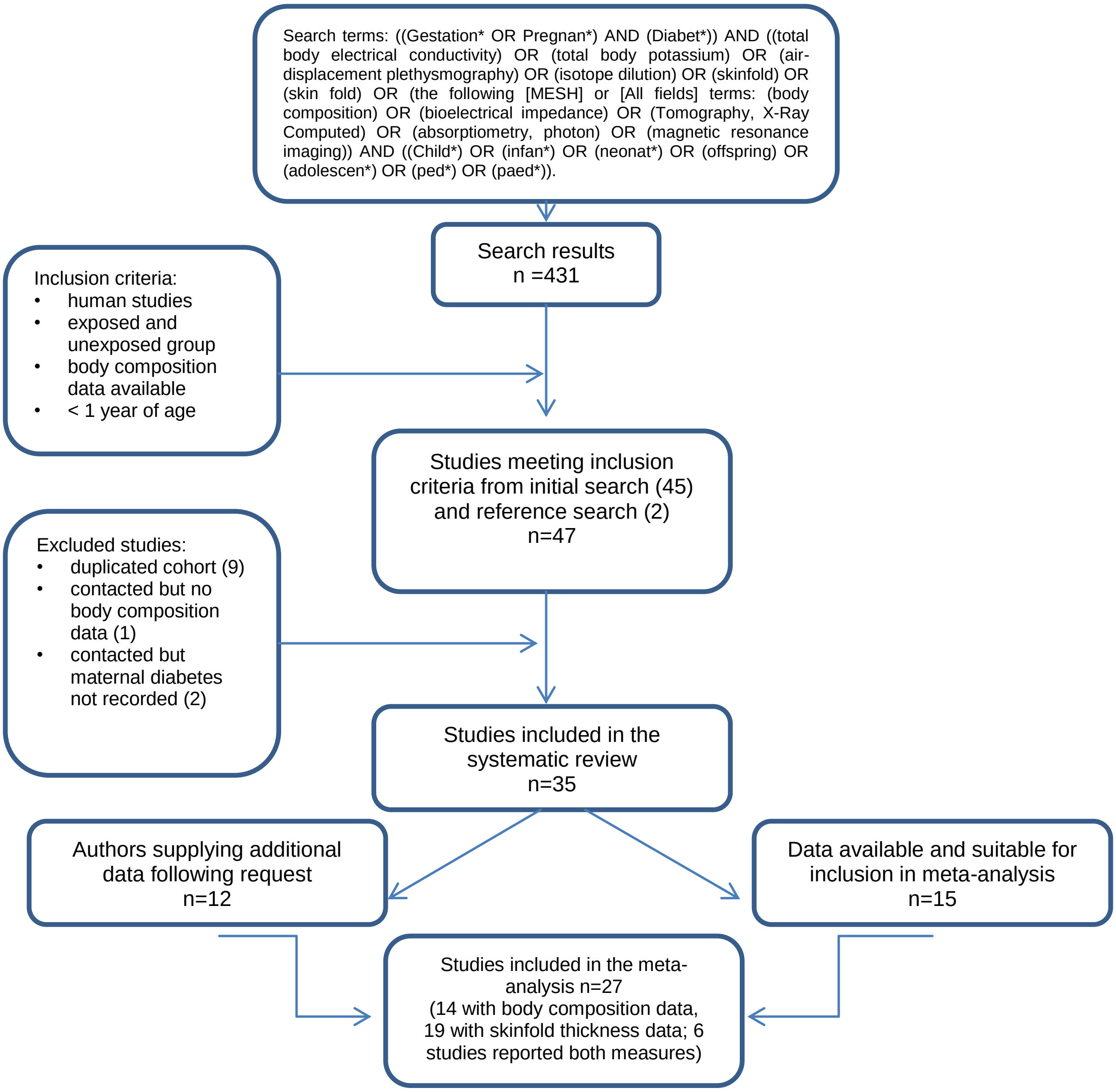

Supplementary figure 1 Flow chart of the search strategy used in this review. The relevant number of papers at each point is given.

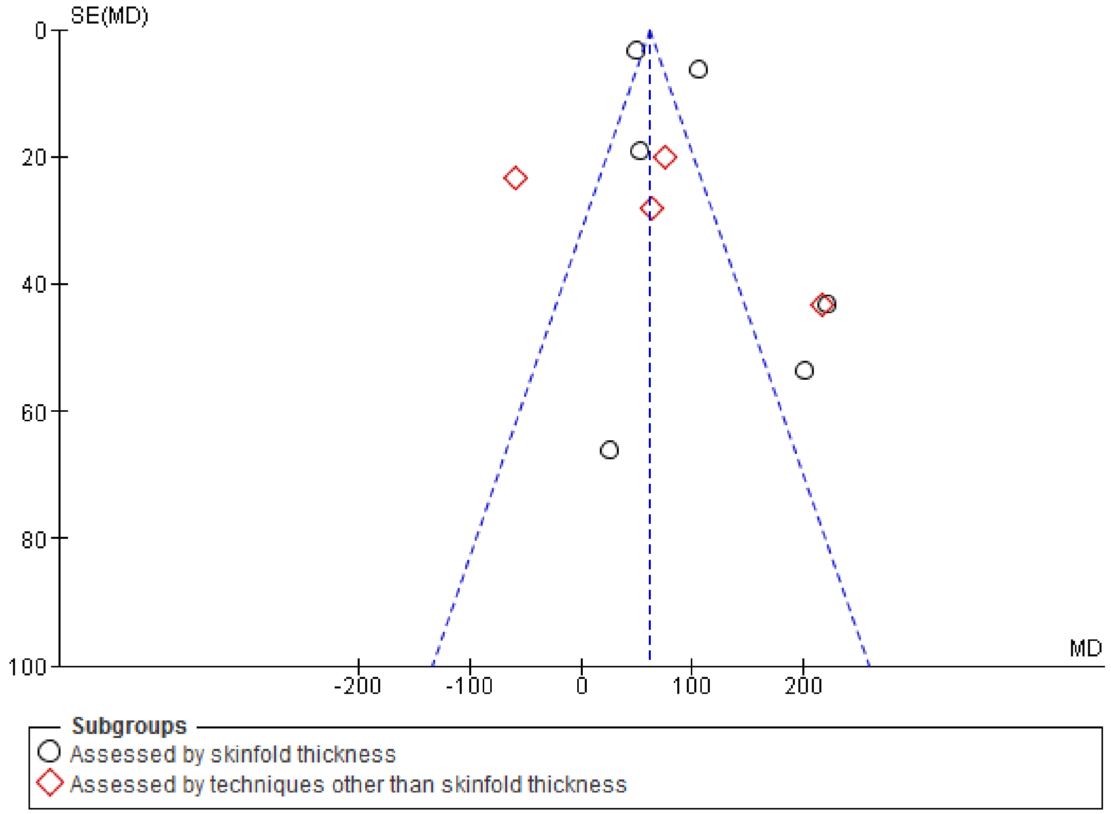

Supplementary figure 2 Funnel plot of studies comparing fat mass (g) in IDM and NIDM (all diabetes types)

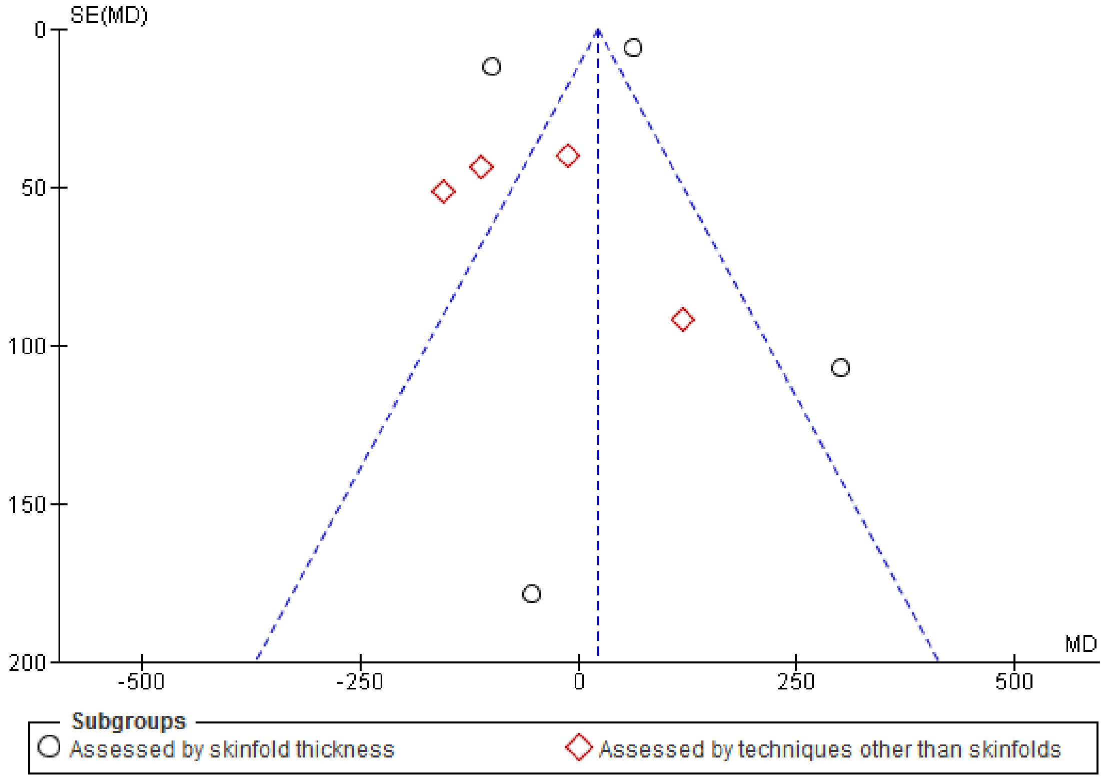

Supplementary figure 3 Funnel plot of studies comparing fat-free mass (g) in IDM and NIDM (all diabetes types)

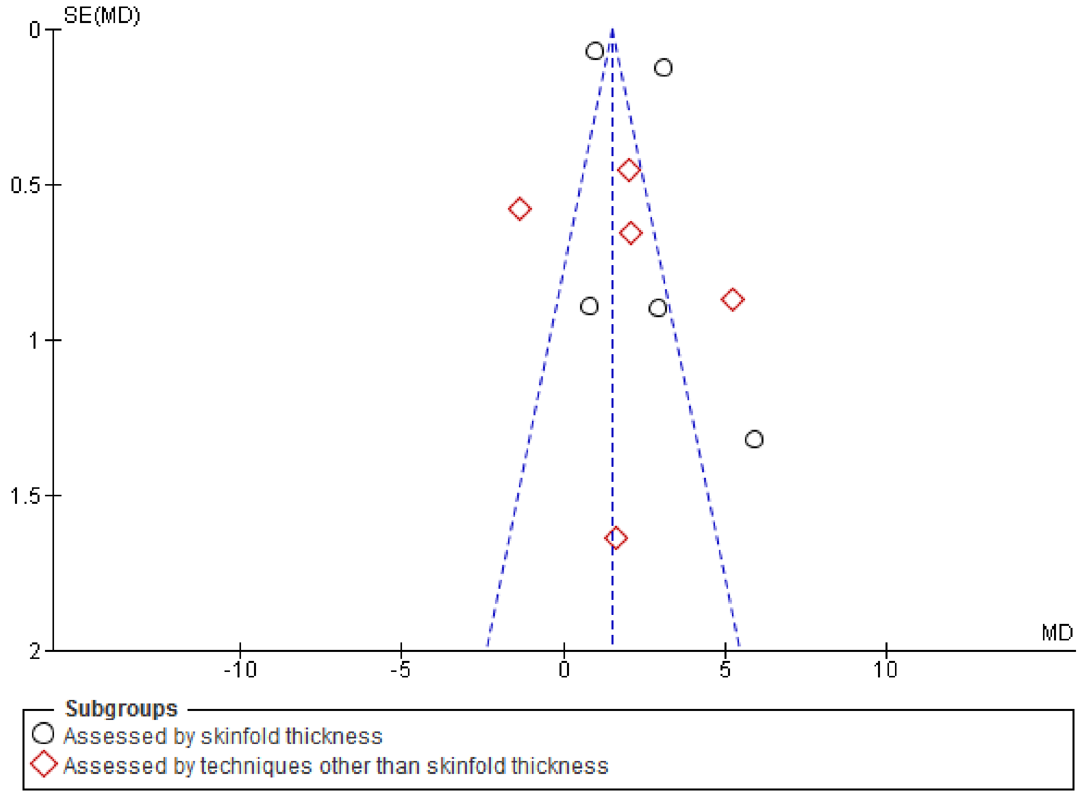

Supplementary figure 4 Funnel plot of studies comparing body fat % in IDM and NIDM (all diabetes types)

Study or Subgroup	IDM Mean [mm]	IDM SD [mm]	IDM Total	Control Mean [mm]	Control SD [mm]	Control Total	Weight	Mean Difference IV, Random, 95% CI [mm]
Aman, 2011	6.7	1.4	28	5.3	1.1	28	3.4%	1.40 [0.74, 2.06]
Brunner, 2013	5.2	0.9	9	4.7	0.9	152	3.8%	0.50 [-0.11, 1.11]
Buhling, 2012	4.6	0.9	30	4.8	1.5	142	5.8%	-0.20 [-0.61, 0.21]
Catalano, 2003	4.7	1.1	195	4.2	1	220	8.4%	0.50 [0.30, 0.70]
Greco, 2003	4.7	0.1	15	4.1	0.1	16	9.6%	0.60 [0.53, 0.67]
Hollingsworth, 1991	4.3	0.9	70	3.8	0.3	211	8.2%	0.50 [0.29, 0.71]
Krishnaveni, 2005	5.1	1.1	41	4.2	0.9	545	6.5%	0.90 [0.55, 1.25]
Metzger, 2009	4.42	0.99	3082	4.08	0.86	16097	9.8%	0.34 [0.30, 0.38]
Nelson, 2007	8	3.1	56	6	2.4	19	1.1%	2.00 [0.65, 3.35]
Rossi, 2000	5.78	1.5	6	4.76	0.28	13	1.3%	1.02 [-0.19, 2.23]
Simmons, 1995	4.5	1	35	4.2	0.8	125	6.3%	0.30 [-0.06, 0.66]
Sletner, 2013	4.71	1.12	65	4.4	1	457	7.3%	0.31 [0.02, 0.60]
Stevenson, 1991	5	1.1	13	4.3	0.8	20	3.2%	0.70 [0.01, 1.39]
Vohr, 1995	4.1	1.1	119	4.1	1.1	143	7.5%	0.00 [-0.27, 0.27]
Westgate, 2006	5.1	1.2	136	4.4	1	61	6.8%	0.70 [0.38, 1.02]
Wurster, 1984	5.9	1.5	10	5	1.5	10	1.2%	0.90 [-0.41, 2.21]
Zhao, 2013	5.2	0.4	160	4.4	0.3	284	9.6%	0.80 [0.73, 0.87]
**Total (95% CI)**			**4070**			**18543**	**100.0%**	**0.52 [0.37, 0.68]**

Heterogeneity: Tau² = 0.06; Chi² = 188.84, df = 16 (P < 0.00001); I² = 92%
Test for overall effect: Z = 6.79 (P < 0.00001)

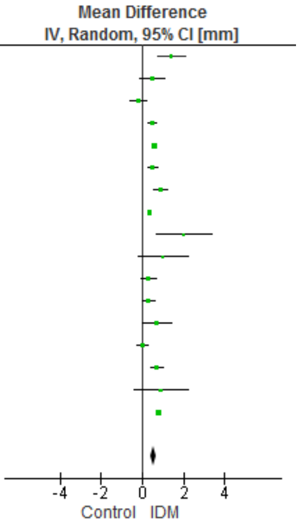

Supplementary figure 5 Forest plot (random effects analysis) comparing triceps skinfold thickness (mm) in IDM and NIDM (all types of diabetes)

Study or Subgroup	IDM Mean [mm]	IDM SD [mm]	IDM Total	Control Mean [mm]	Control SD [mm]	Control Total	Weight	Mean Difference IV, Random, 95% CI [mm]
Aman, 2011	6.3	1.8	28	4.8	1.1	28	4.7%	1.50 [0.72, 2.28]
Brunner, 2013	4.7	0.8	9	4.5	1	152	6.2%	0.20 [-0.35, 0.75]
Buhling, 2012	4.3	1.1	12	4.1	0.97	13	4.5%	0.20 [-0.62, 1.02]
Catalano, 2003	5.1	1.4	195	4.6	1.2	220	8.0%	0.50 [0.25, 0.75]
Greco, 2003	4.7	0.1	15	3.8	0.1	16	8.7%	0.90 [0.83, 0.97]
Hollingsworth, 1991	5	1.1	70	4	0.4	211	8.0%	1.00 [0.74, 1.26]
Krishnaveni, 2005	5.3	1.1	41	4.4	0.9	545	7.5%	0.90 [0.55, 1.25]
Metzger, 2009	4.59	1.13	3082	4.17	0.95	16097	8.7%	0.42 [0.38, 0.46]
Nelson, 2007	7.4	2.1	56	5.6	2	19	3.4%	1.80 [0.75, 2.85]
Rossi, 2000	5.78	1.25	6	4.47	2.2	13	2.0%	1.31 [-0.25, 2.87]
Simmons, 1995	6.3	2	35	5.3	1.3	125	5.2%	1.00 [0.30, 1.70]
Sletner, 2013	4.84	1.32	65	4.26	1.02	457	7.6%	0.58 [0.25, 0.91]
Vohr, 1995	4.7	1.5	119	4.6	1.3	143	7.5%	0.10 [-0.24, 0.44]
Westgate, 2006	5.6	1.7	136	4.4	1	61	7.3%	1.20 [0.82, 1.58]
Wurster, 1984	6.6	1.9	10	5.2	1.6	10	2.0%	1.40 [-0.14, 2.94]
Zhao, 2013	5.2	0.3	160	4	0.2	284	8.7%	1.20 [1.15, 1.25]
**Total (95% CI)**			**4039**			**18394**	**100.0%**	**0.81 [0.56, 1.05]**

Heterogeneity: Tau² = 0.19; Chi² = 579.15, df = 15 (P < 0.00001); I² = 97%
Test for overall effect: Z = 6.33 (P < 0.00001)

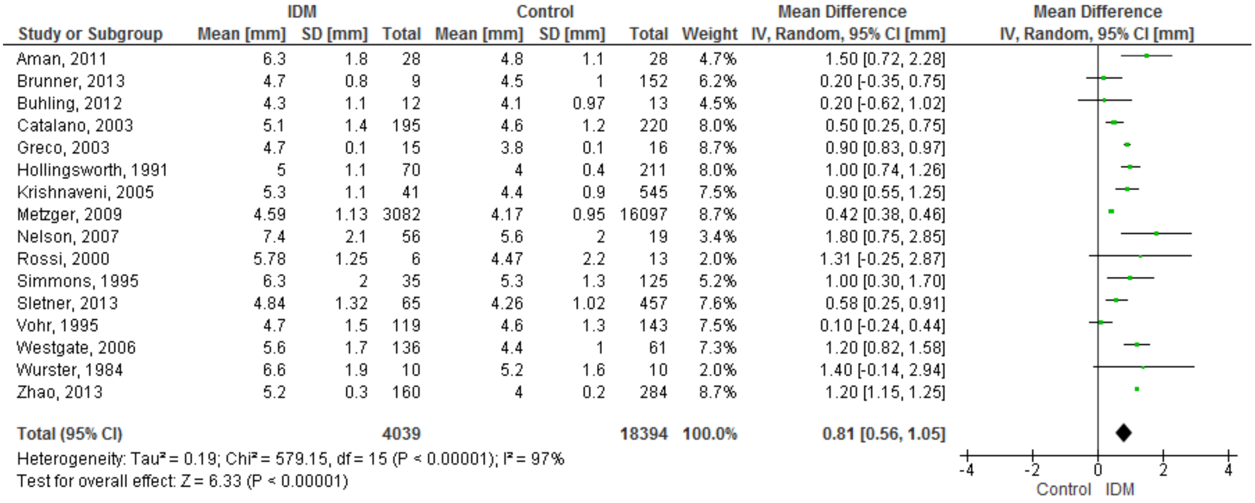

Supplementary figure 6 Forest plot (random effects analysis) comparing subscapular skinfold thickness (mm) in IDM and NIDM (all types of diabetes)

Study or Subgroup	IDM Mean [grams]	IDM SD [grams]	IDM Total	Control Mean [grams]	Control SD [grams]	Control Total	Weight	Mean Difference IV, Random, 95% CI [grams]
**13.1.1 GDM**								
Aman, 2011	700	200	10	500	200	28	4.0%	200.00 [55.59, 344.41]
Au, 2013	272	180	67	331	180	532	11.3%	-59.00 [-104.73, -13.27]
Brunner, 2013	509	195	9	484	142	152	4.7%	25.00 [-104.38, 154.38]
Catalano, 2003	436	206	195	362	198	220	12.0%	74.00 [35.01, 112.99]
Enzi, 1980	553	202	17	386	90.7	17	6.0%	167.00 [61.74, 272.26]
Lingwood, 2011	410	170	84	350	162	77	10.8%	60.00 [8.71, 111.29]
Metzger, 2009	424	177	3082	375	159	16097	14.2%	49.00 [42.29, 55.71]
Schafer-Graf, 2011	433	171	150	381	179	190	12.1%	52.00 [14.63, 89.37]
Zhao, 2013	585	59	160	480	74	284	14.0%	105.00 [92.44, 117.56]
**Subtotal (95% CI)**			**3774**			**17597**	**89.0%**	**61.69 [29.09, 94.29]**
Heterogeneity: Tau² = 1635.80; Chi² = 94.19, df = 8 (P < 0.00001); I² = 92%								
Test for overall effect: Z = 3.71 (P = 0.0002)								
**13.1.2 T1D**								
Aman, 2011	700	200	18	500	200	28	5.2%	200.00 [81.58, 318.42]
Enzi, 1980	718	144	8	386	91	17	5.8%	332.00 [223.24, 440.76]
**Subtotal (95% CI)**			**26**			**45**	**11.0%**	**268.17 [138.88, 397.45]**
Heterogeneity: Tau² = 5347.04; Chi² = 2.59, df = 1 (P = 0.11); I² = 61%								
Test for overall effect: Z = 4.07 (P < 0.0001)								
**Total (95% CI)**			**3800**			**17642**	**100.0%**	**85.44 [51.43, 119.45]**

Heterogeneity: Tau² = 2115.52; Chi² = 123.44, df = 10 (P < 0.00001); I² = 92%
Test for overall effect: Z = 4.92 (P < 0.00001)
Test for subgroup differences: Chi² = 9.21, df = 1 (P = 0.002), I² = 89.1%

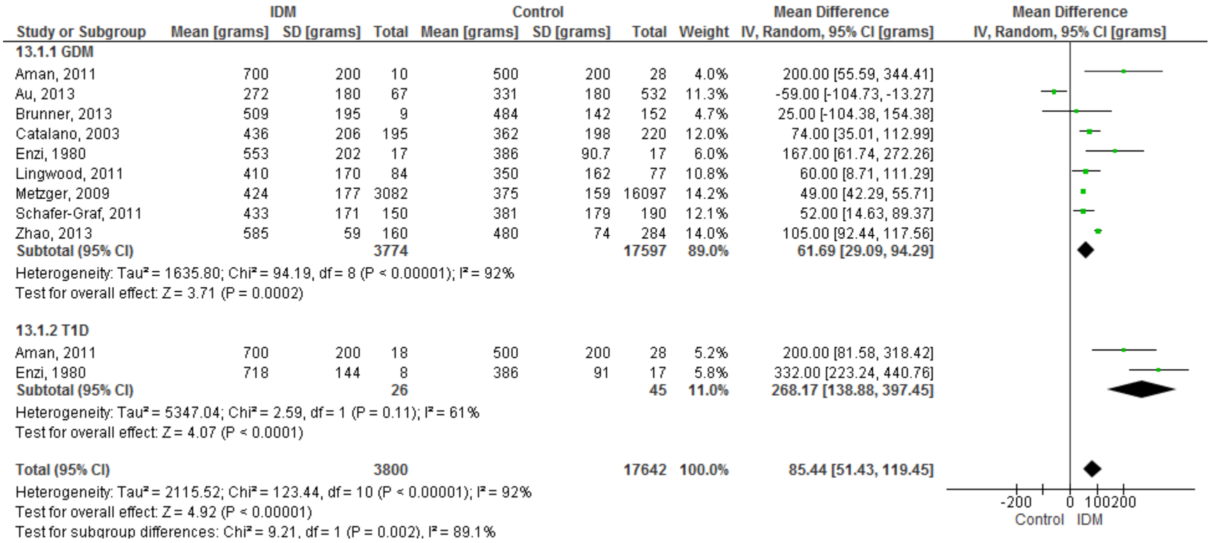

Supplementary figure 7 Forest plot (random effects analysis) comparing fat mass (g) in infants of mothers with and without diabetes with subgroup analysis by maternal diabetes type (GDM and T1D)

Study or Subgroup	IDM Mean	IDM SD	IDM Total	Control Mean	Control SD	Control Total	Weight	Mean Difference IV, Random, 95% CI
**13.2.1 GDM**								
Aman, 2011	17	3.2	10	13.5	3.5	28	7.5%	3.50 [1.13, 5.87]
Au, 2013	7.9	4.5	67	9.3	4.3	532	10.7%	-1.40 [-2.54, -0.26]
Brumbaugh, 2013	14.7	3	12	13.1	5	13	5.6%	1.60 [-1.60, 4.80]
Brunner, 2013	14.5	2.6	9	13.7	2.8	152	9.1%	0.80 [-0.96, 2.56]
Catalano, 2003	12.4	4.6	195	10.4	4.6	220	11.3%	2.00 [1.11, 2.89]
Enzi, 1980	17.1	7	17	12.2	2.1	17	5.1%	4.90 [1.43, 8.37]
Lingwood, 2011	12.1	4.3	84	10.05	4.05	77	10.3%	2.05 [0.76, 3.34]
Metzger, 2009	12.2	3.7	3082	11.2	3.53	16097	12.3%	1.00 [0.86, 1.14]
Zhao, 2013	17.8	0.8	160	14.7	1.9	284	12.2%	3.10 [2.85, 3.35]
**Subtotal (95% CI)**			**3636**			**17420**	**84.1%**	**1.73 [0.65, 2.81]**
Heterogeneity: Tau² = 2.03; Chi² = 234.98, df = 8 (P < 0.00001); I² = 97%								
Test for overall effect: Z = 3.14 (P = 0.002)								
**13.2.2 T1D**								
Aman, 2011	16.1	3.2	18	13.5	3.5	28	8.5%	2.60 [0.63, 4.57]
Enzi, 1980	20.3	3.1	8	12.2	2.1	17	7.5%	8.10 [5.73, 10.47]
**Subtotal (95% CI)**			**26**			**45**	**15.9%**	**5.31 [-0.08, 10.70]**
Heterogeneity: Tau² = 13.89; Chi² = 12.26, df = 1 (P = 0.0005); I² = 92%								
Test for overall effect: Z = 1.93 (P = 0.05)								
**Total (95% CI)**			**3662**			**17465**	**100.0%**	**2.29 [1.26, 3.32]**
Heterogeneity: Tau² = 2.24; Chi² = 266.03, df = 10 (P < 0.00001); I² = 96%								
Test for overall effect: Z = 4.36 (P < 0.0001)								
Test for subgroup differences: Chi² = 1.63, df = 1 (P = 0.20), I² = 38.6%								

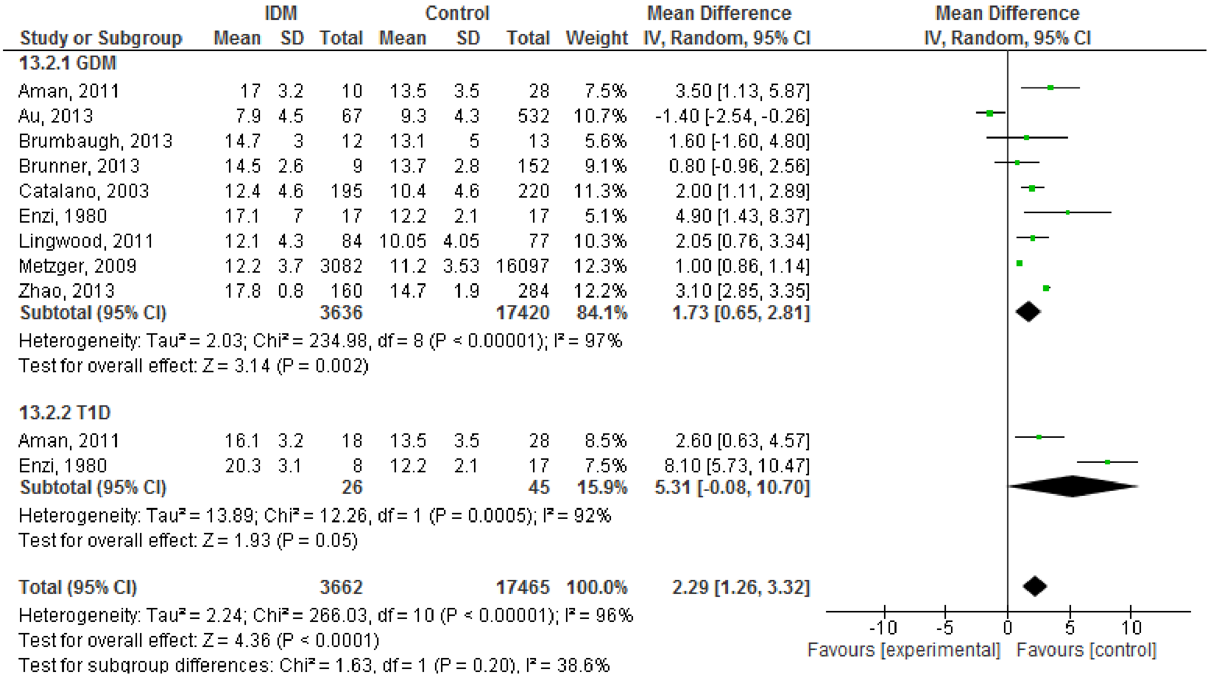

Supplementary figure 8 Forest plot (random effects analysis) comparing body fat % in infants of mothers with and without diabetes with subgroup analysis by maternal diabetes type (GDM and T1D)

Study or Subgroup	IDM Mean	IDM SD	IDM Total	Control Mean	Control SD	Control Total	Weight	Mean Difference IV, Random, 95% CI
Aman, 2011	3,400	400	10	3,100	400	28	6.8%	300.00 [11.18, 588.82]
Au, 2013	2,846	338	67	2,959	342	532	16.8%	-113.00 [-198.99, -27.01]
Brunner, 2013	2,933	528	9	2,988	350	152	5.2%	-55.00 [-404.41, 294.41]
Catalano, 2003	2,962	405	195	2,975	408	220	17.3%	-13.00 [-91.34, 65.34]
Lingwood, 2011	2,961	444	84	3,045	320	77	14.9%	-84.00 [-202.84, 34.84]
Metzger, 2009	2,928	334	3082	2,866	311	16097	19.6%	62.00 [49.27, 74.73]
Zhao, 2013	2,685	127	160	2,784	109	284	19.4%	-99.00 [-122.41, -75.59]
**Total (95% CI)**			**3607**			**17390**	**100.0%**	**-23.33 [-116.41, 69.74]**
Heterogeneity: Tau² = 11470.78; Chi² = 157.59, df = 6 (P < 0.00001); I² = 96%								
Test for overall effect: Z = 0.49 (P = 0.62)								

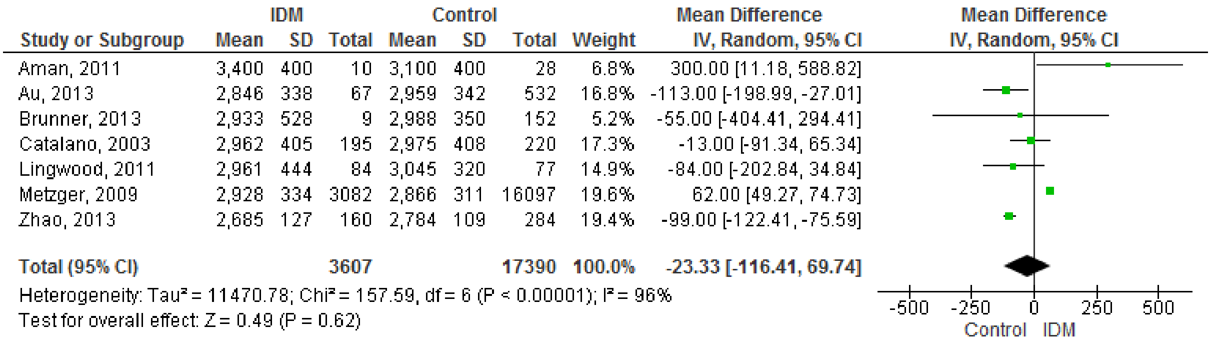

Supplementary figure 9 Forest plot (random effects analysis) comparing fat-free mass (g) in infants of mothers with and without GDM

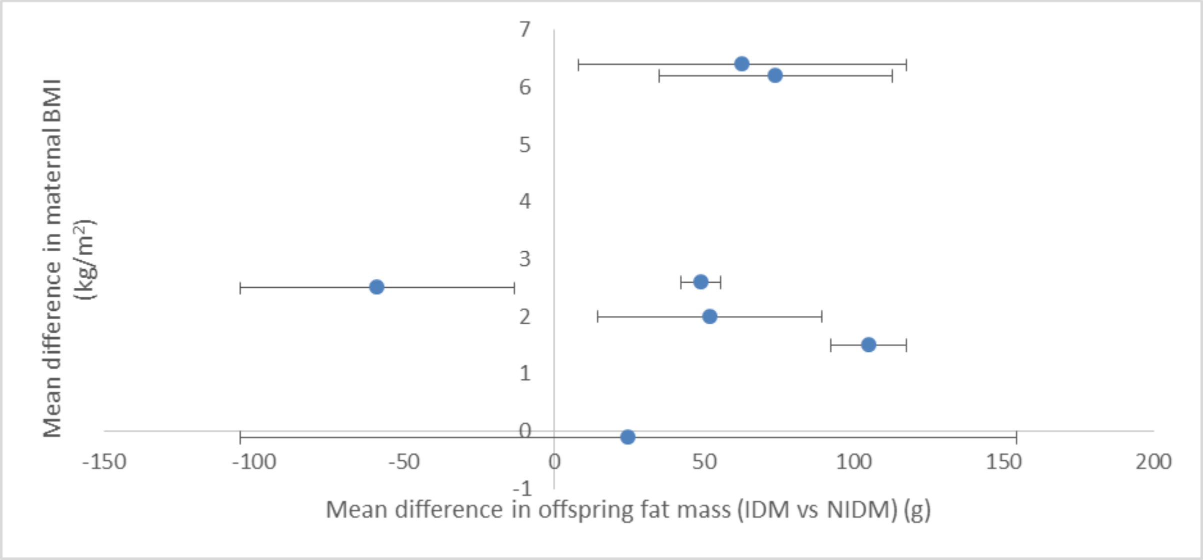

Supplementary figure 10 Mean difference in maternal BMI between mothers with and without diabetes in each study against mean difference in infant fat mass, showing no evidence of increasing fat mass with increasing maternal BMI.

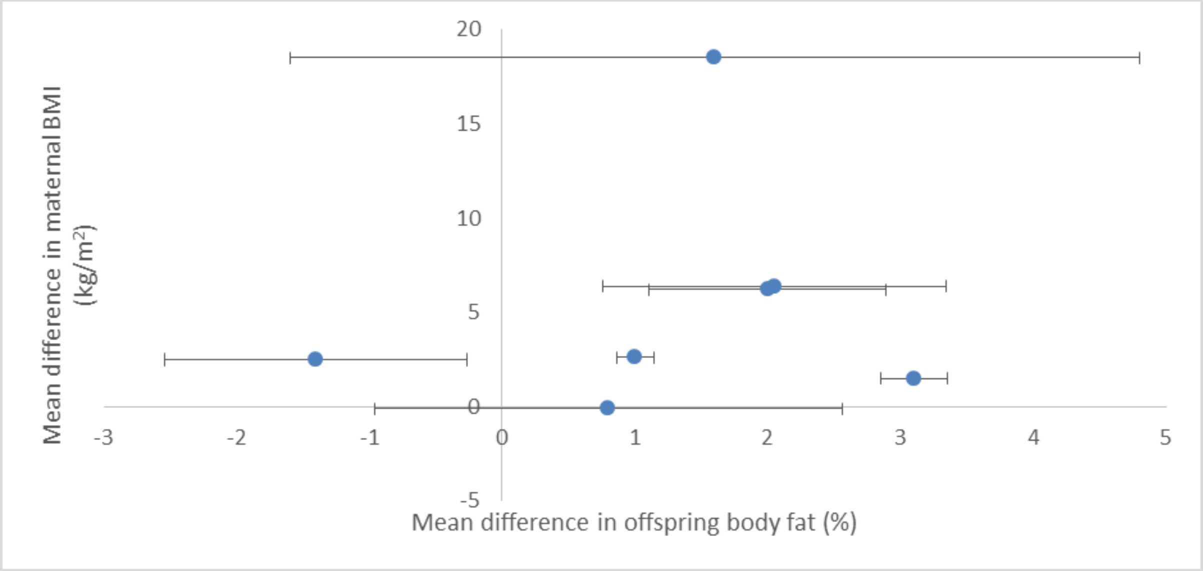

Supplementary figure 11 Mean difference in maternal BMI between mothers with and without diabetes in each study against mean difference in infant body fat %, showing no evidence of increasing body fat % with increasing maternal BMI.
