# Supplementary material for: Diabetes in pregnancy and infant adiposity: systematic review and meta-analysis
Source: Arch Dis Child Fetal Neonatal Ed. 2016 May 26;102(1):F65–72. doi: 10.1136/archdischild-2015-309750 (PMC5256410; doi:10.1136/archdischild-2015-309750)
Supplement: Supplementary data [file fetalneonatal-2015-309750supp2.pdf]

Modified Newcastle-Ottawa Quality Assessment Scale Scoring Sheet:
Maternal diabetes and adiposity in infancy – meta-analysis

First Author:
Year of Publication:
Initials of Scorer and Date:

Section		Tick	Star score
**Selection**			
**1) Representativeness of the exposed cohort**			
A: Truly representative (regional/national)	A	*	
B: Somewhat representative (multi/single centre)	B	*	
C: Selective group (exclusion criteria)	C		
D: Not described	D		
**2) Selection of the non exposed cohort**			
A: Same community as exposed	A	*	
B: Different source	B		
C: Not described	C		
**3) Ascertainment of exposure (maternal diabetes status)**			
A: Secure record (medical notes/register)	A	*	
B: Structured interview	B	*	
C: Written self report	C		
D: Not described	D		
**Section Subtotal Score (out of 3):**			
**Outcome**			
**1) Assessment of outcome**			
A: Independent blind assessment	A	*	
B: Record linkage	B	*	
C: Self report	C		
D: Non-blind assessment	D		
E: No description	E		
**2A - *For cohort studies:* Adequacy of follow up of cohorts**			
A: Complete	A	*	
B: >80% follow up or well described lost group	B	*	
C: <80% follow up and no description	C		
D: No statement	D		
**2B - *For case control studies:* Non-Response rate**			
A: Same for both groups	A	*	
B: Non-respondents described	B		
C: Rate different and no designation	C		
**Section Subtotal Score (out of 2):**			
**TOTAL SCORE:**			
